# Engineered niches support the development of human dendritic cells in humanized mice

**DOI:** 10.1038/s41467-020-15937-y

**Published:** 2020-04-28

**Authors:** Giorgio Anselmi, Kristine Vaivode, Charles-Antoine Dutertre, Pierre Bourdely, Yoann Missolo-Koussou, Evan Newell, Oliver Hickman, Kristie Wood, Alka Saxena, Julie Helft, Florent Ginhoux, Pierre Guermonprez

**Affiliations:** 1 Centre for Inflammation Biology and Cancer Immunology, The Peter Gorer Department of Immmunobiology, King’s College London, London, UK; 2 Cancer Research UK, King’s Health Partners Cancer Centre, King’s College London, London, UK; 3 Singapore Immunology Network (SIgN), A*STAR, Singapore, Singapore; 4 Paris-Sciences-Lettres University, Institut Curie Research Center, INSERM U932 & SiRIC, Translational Immunotherapy Team, Paris, France; 5 National Institute of Health Research Biomedical Research Centre at Guy’s and St Thomas’ Hospital and King’s College London, London, UK; 6 Université de Paris, Centre for Inflammation Research, CNRS ERL8252, INSERM1149, Paris, France; 7 Present Address: MRC Molecular Hematology Unit, MRC Weatherall Institute of Molecular Medicine, Radcliffe Department of Medicine, University of Oxford, Oxford, UK; 8 Present Address: Drug Target Discovery Team, Division of Breast Cancer Research, Institute of Cancer Research, London, UK; 9 Present Address: Labcyte Ltd, Norton Canes, Cannock, Staffordshire, UK

**Keywords:** Conventional dendritic cells, Haematopoietic stem cells, Stem-cell differentiation, Stem-cell niche

## Abstract

Classical dendritic cells (cDCs) are rare sentinel cells specialized in the regulation of adaptive immunity. Modeling cDC development is crucial to study cDCs and harness their therapeutic potential. Here we address whether cDCs could differentiate in response to trophic cues delivered by mesenchymal components of the hematopoietic niche. We find that mesenchymal stromal cells engineered to express membrane-bound FLT3L and stem cell factor (SCF) together with CXCL12 induce the specification of human cDCs from CD34^+^ hematopoietic stem and progenitor cells (HSPCs). Engraftment of engineered mesenchymal stromal cells (eMSCs) together with CD34^+^ HSPCs creates an in vivo synthetic niche in the dermis of immunodeficient mice driving the differentiation of cDCs and CD123^+^AXL^+^CD327^+^ pre/AS-DCs. cDC2s generated in vivo display higher levels of resemblance with human blood cDCs unattained by in vitro-generated subsets. Altogether, eMSCs provide a unique platform recapitulating the full spectrum of cDC subsets enabling their functional characterization in vivo.

## Abstract (web summary)

Classical human dendritic cells (cDCs) are rare sentinel cells specialized in regulating adaptive immunity. Here, the authors show that expression of membrane bound FLT3L, along with stem cell factor (SCF) and CXCL12 in stromal cells induces specification of pre/AS-DCs, type 1 and type2 cDC from haematopoietic stem cells.

## Introduction

Classical human dendritic cells (cDCs) are sentinels of the immune system with a unique ability to regulate the function of T lymphocytes^1^. Dendritic cells (DCs) can induce immune tolerance^2^ or drive the development of immunity^3^.

The analysis of blood circulating subsets has revealed that cDCs consist in two major subtypes: CD141^+^XCR1^+^Clec9A^+^ DCs (cDC1) and CD1c^+^CD11c^+^CD172a (SIRPα)^+^ DCs (cDC2s)^4–6^. Both cDC1s and cDC2s arise from a bone marrow committed progenitor^7^ or from early IRF8^+^ multipotent progenitors^8,9^, which generate a common circulating precursor^10^ that progressively diverge in pre-cDC1s and pre-cDC2s^10–12^. Type 1 DCs are conserved between mouse and human, and they share the expression of specific surface markers such as Clec9A^13^ and XCR1^5^, as well as the transcription factor (TF) IRF8, which is essential for the development of murine cDC1s^4–6,13–15^. Conversely, human CD1c^+^ type 2 DCs have been shown to express the IRF4 TF^16^, which controls the development of their phenotypic equivalent in the mouse model^16,17^. This rather simple picture is complicated by the diversity of CD1c^+^ cells, which encompass migratory DCs and CD14^int^ inflammatory DCs recruited during inflammation^18,19^. More recently, unbiased approaches have uncovered a deeper complexity in the DC network with the identification of two types of cDC2s with distinct transcriptional profiles and the identification of AXL^+^CD11c^+^CD1c^+^ cells, which have been proposed to act as a precursor for cDCs^12,20^.

Human hematopoietic progenitors reside in the stem cells niche of the bone marrow. Genetic studies in the murine model identified three essential factors supporting HSPCs homeostasis: the membrane-bound form of stem cell factor (SCF/KITL)^21,22^, the C-X-C motive chemokine 12 (CXCL12)^23,24^ and thrombopoietin (TPO)^25,26^. In the bone marrow, perivascular mesenchymal stromal cells have been described as the main source of SCF and other niche factors^27^. At steady state, Flt3-ligand (FLT3L) is delivered as a membrane-bound precursor expressed on radio-resistant stromal cells^28–30^. After egressing from the bone marrow, DC precursors circulate in the blood and seed the peripheral tissues^31^. In the lymph node, stromal fibroblastic reticular cells provide FLT3L^32^ and FLT3-dependent proliferation of cDC in periphery is required for their maintenance^33,34^.

Modeling the development of cDCs in culture systems is instrumental to better understand their ontogeny and define their immunological function. Pioneer work from Banchereau et al.^35^ have identified that granulocyte–macrophage colony-stimulating factor (GM-CSF) and tumor necrosis factor-α (TNF-α) cooperate to produce CD1a^+^ cells with features of Langerhans cells from CD34^+^ hematopoietic stem and progenitor cells (HSPCs). Sallusto et al.^36^ have shown that GM-CSF and interleukin (IL)-4 induce the differentiation of CD1c^+^CD1a^+^ inflammatory DCs from CD14 monocytes. More recent work has demonstrated that FLT3L (with TPO or with SCF/KITL, GM-CSF, and IL-4) is instrumental in generating CD141^+^ cDC1s aligning phenotypically and functionally with cDC1s^7,8,37–39^. This is in line with the crucial role of FLT3L, engaging the Flt3 receptor tyrosine kinase^40,41^ in controlling DC homeostasis both in mice^31,33,42,43^ and humans^10,28,44,45^. Moreover, the activation of Notch signaling pathway has been shown to further improve the in vitro differentiation of both human and mouse cDC1s^46,47^. Despite the successes in modeling cDC1 differentiation in vitro, CD1c^+^ cells found in culture of CD34^+^ HSPCs either align poorly with bona fide blood circulating cDC2s^38^ or were not extensively characterized^46,47^.

Recapitulating human cDC development in vivo has the potential to greatly improve our understanding of DC biology and facilitate its translational applications. Human cDCs have been found in stably reconstituted humanized mice treated with supraphysiological concentration of human FLT3L^28,48,49^. However, the generated CD11c^+^CD141^+^ and CD11c^+^CD1c^+^ cells were poorly characterized and their dissemination to peripheral tissues has rarely been assessed^50^.

Here we aimed at modeling human cDC development by providing physiological factors associated to hematopoietic niches. We found that engineered mesenchymal stromal cells (eMSCs) expressing a combination of membrane-bound FLT3L and SCF/KITL together with CXCL12 provide a scaffold for human cDC differentiation. Engraftment of eMSCs along with CD34^+^ HSPCs leads to the local development of cDCs in immunodeficient mice. This in vivo system recapitulates the differentiation of not only pDCs, cDC1s, and cDC2s but also pre/AS-DCs, and reaches an unmatched level of similarity of the generated cDC2s with their blood counterparts.

## Results

### Transmembrane FLT3L drives human DC differentiation in vitro

We hypothesized that the interaction of hematopoietic progenitors with membrane-bound factors expressed by stromal cells of the niche would promote the specification of the cDC lineage.

**Fig. 1:**
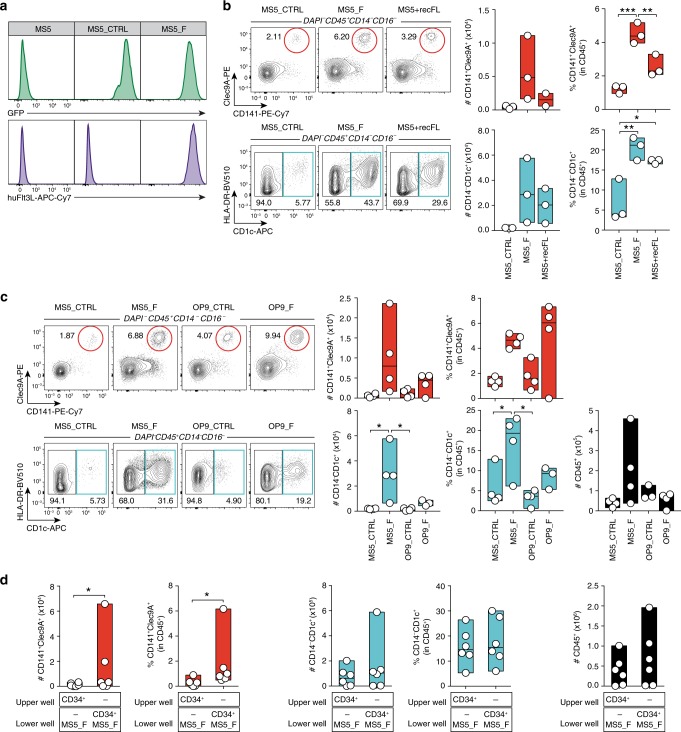
Transmembrane FLT3L drives human DC differentiation in vitro. **a** Expression of membrane-bound FLT3L in mouse bone marrow-derived stromal cells engineered to express human FLT3L (MS5_F) and control (MS5_CTRL). **b** Human cDC subsets differentiated in vitro from CD34^+^ cord blood-derived HSPCs cultured with MS5 expressing membrane-bound FLT3L (MS5_F) or MS5 supplemented with recombinant human FLT3L (MS5+recFL) at day 15 (*n* = 3 donors in one experiment). *p < 0.05, **p < 0.01, ***p < 0.001, one-way ANOVA test with Tukey’s multiple comparisons. **c** Representative flow cytometry plots and quantification of human cDC subsets differentiated in vitro from cord blood-derived CD34^+^ progenitors in culture with mouse stromal cell lines MS5 and OP9 expressing human FLT3L (MS5_F and OP9_F) at day 15 (*n* = 4 donors in one experiment). *p < 0.05, one-way ANOVA test with Tukey’s multiple comparisons). **d** Absolute number and frequency of CD141^+^Clec9A^+^ and CD14^−^CD1c^+^ human cDCs differentiated from CD34^+^ HSPCs in direct contact (lower well) or physically separated (upper well) from engineered MS5_F. DC differentiation was assessed at day 15 by flow cytometry (*n* = 6 donors in three independent experiments). *p < 0.05, two-tailed paired Student’s *t*-test. Data are presented as floating bars ranging from min to max and line represents median (**b**–**d**).

To test this, we engineered a bone marrow-derived murine mesenchymal cell line (MS5)^7,51^ to stably and homogeneously express the transmembrane form of human FLT3L (MS5_F) as probed by flow cytometry (Fig. 1a). Co-culture of MS5_F with CD34^+^ HSPCs drives the appearance of cDC1-like Clec9A^+^CD141^+^ and cDC2-like CD14^−^CD1c^+^ cells. Importantly, MS5_F is more efficient than recombinant soluble FLT3L (MS5 + recFL) in generating cDC-like cells (Fig. 1b).

In contrast, OP9^52^ hematogenic stromal cells stably expressing membrane-bound FLT3L (OP9_F) were less efficient than MS5_F in driving cDC differentiation (Fig. 1c). Besides, MS5_F also promoted the appearance of CD123^+^CD303/4^+^ cells resembling either pDCs or pre/AS-DCs^12,20^ (Supplementary Fig. 1a, b).

Next, we wanted to test whether cell-to-cell interactions mediate the differentiation of cDCs driven by FLT3L-expressing MS5 stromal cells. Using transwell permeable to soluble factors but preventing cognate interactions, we found that direct contact is required to support efficiently cDC differentiation (Fig. 1d).

Collectively, these data show that membrane FLT3L expression in stromal cells provide an improved platform to trigger the differentiation of cDC-like cells from CD34^+^ HSPCs in vitro via cell-to-cell contact.

### CXCL12 and SCF improve FLT3L-driven DC differentiation

Next, we sought to improve the efficiency of cDC production in MS5_F by co-expressing additional niche factors. We focused on SCF, CXCL12, and TPO because of their essential role in supporting HSPCs maintenance in the bone marrow niche^21,23–26,53^. SCF had also been extensively used in previously published DC culture protocols^7,38,39,54^.

To this end, we generated a collection of MS5 stromal cells stably expressing either one, two, three, or four human factors by combining CXCL12, TPO, and membrane-bound SCF/KITL, with or without membrane-bound FLT3L (Supplementary Fig. 2a).

We screened this collection of eMSC lines based on their ability to support human cDC differentiation from cord blood-derived CD34^+^ HSPCs.

**Fig. 2:**
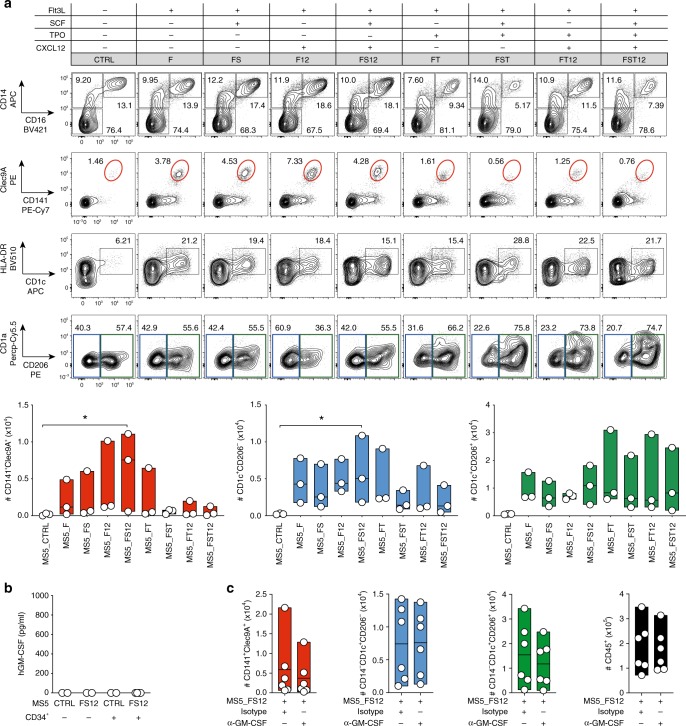
CXCL12 and SCF improve FLT3L-driven DC differentiation. **a** Representative FACS plots and absolute number of CD141^+^Clec9A^+^, CD1c^+^CD206^−^, and CD1c^+^CD206^+^ cells generated from CD34^+^ HSPCs cultured with MS5 expressing human FLT3L (MS5_F) in combination with human SCF (S), TPO (T), and CXCL12 (12). Day 15 flow cytometry analysis of *n* = 3 cord blood donors in three independent experiments. *p < 0.05, one-way ANOVA test with Tukey’s multiple comparisons. **b** ELISA detecting human GM-CSF in the supernatant of CD34^+^ HSPCs cultured with engineered MS5 expressing human FLT3L, SCF and CXCL12 (MS5_FS12) at day 15 for *n* = 2 (MS5_CTRL ± CD34^+^ HSPC and MS5_FS12 only) and *n* = 4 (MS5_FS12 + CD34^+^ HSPC) independent donors. **c** Absolute number of human DC subsets generated in vitro from CD34^+^ HSPC using MS5_FS12 stromal cells in the presence of human GM-CSF neutralizing antibody as compared to isotype control (*n* = 6 independent donors in two experiments). Data are presented as floating bars ranging from min to max and line represents median **a**, **c** or as b**a**r graphs with mean ± SEM (**b**).

At day 15, only FLT3L-expressing eMSCs successfully supported the differentiation of CD141^+^Clec9A^+^ and CD14^−^CD1c^+^ cells (Fig. 2a and Supplementary Figs. 2b and  3a). We conclude that FLT3L is necessary for the differentiation of cDCs using eMSCs. Besides, optimal cDC production was obtained in cultures containing eMSC co-expressing membrane-bound SCF and CXCL12 together with FLT3L (MS5_FS12) (Fig. 2a), whereas no difference was observed for CD14^+^CD16^−^ monocytes and CD14^+^CD16^+^ macrophages as compared to MS5_CTRL (Supplementary Fig. 3b).

Furthermore, we noticed that in vitro differentiated CD14^−^CD1c^+^ cDC2-like cells were heterogeneous for the expression of the mannose receptor CD206 (Fig. 2a). Circulating blood cDC2s do not generally express CD206 (Supplementary Fig. 3c), whereas CD206 is a marker of skin and migratory cDC2^19,55^.

Most of the previously described protocols to generate human DC-like cells in vitro from both CD14^+^ monocytes and CD34^+^ HSPCs made an extensive use of the cytokine GM-CSF^7,8,36,38,39,54^, with one exception^47^. As we did not include GM-CSF in our protocol, we wanted to assess whether human GM-CSF was spontaneously produced in CD34^+^ cultures. We could not detect any GM-CSF from co-culture supernatant (Fig. 2b). Accordingly, GM-CSF-blocking antibody did not impact the generation of cDCs driven by MS5_FS12 (Fig. 2c). We conclude that GM-CSF is dispensable for the generation of cDCs in vitro, as previously reported in both mouse^56,57^ and human^47^.

**Fig. 3:**
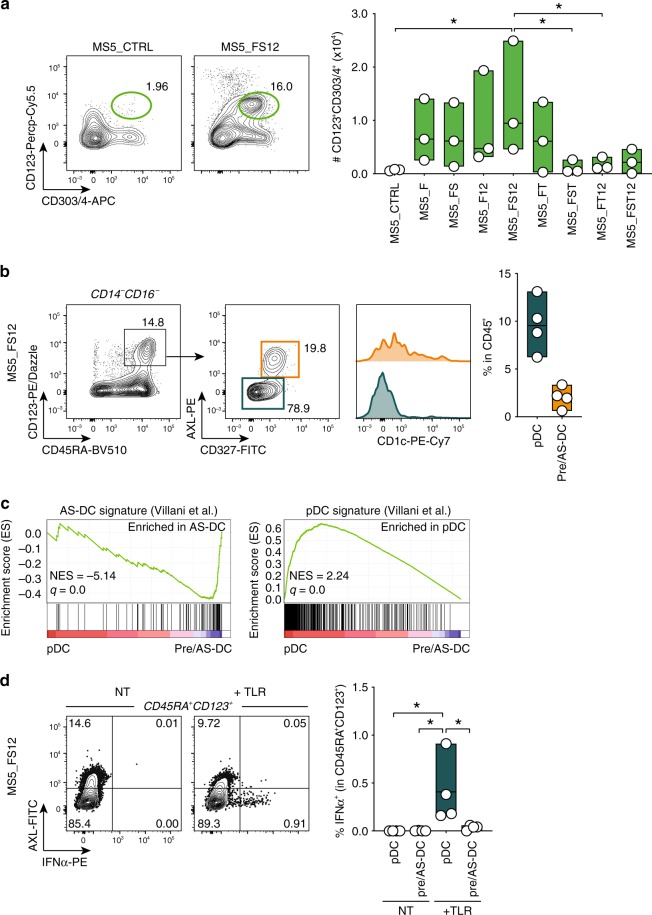
MS5_FS12 stromal cells support pDC and pre/AS-DC development in vitro. **a** Representative FACS plots and absolute number of CD123^+^CD303/4^+^ cells generated in vitro from CD34^+^ HSPCs co-cultured with MS5 expressing human FLT3L (MS5_F) in combination with human SCF (S), TPO (T), and CXCL12 (12). Day 15 flow cytometry analysis of *n* = 3 cord blood donors in three independent experiments. *p < 0.05, one-way ANOVA test with Tukey’s multiple comparisons. **b** Gating strategy used to identify AXL^−^CD327^lo/−^ pDC and AXL^+^CD327^+^ pre^/^AS-DC within CD123^+^CD45RA^+^ cells generated in vitro using MS5_FS12. Graph illustrates the frequency of each subset in CD45^+^ cells (*n* = 4 cord blood donors). **c** GSEA comparing in vitro-differentiated pDC and pre/AS-DC using published human pDC and AS-DC gene signatures^20^ (FDR false detection rate, NES normalized enrichment score). Statistical significance is defined by the FDR *q*-value calculated by the GSEA software (www.broad.mit.edu/gsea) using default parameters. **d** Intracellular flow cytometry analysis of IFNα production in pDC and pre/AS-DC in response to 16 h of TLR stimulation (lipopolysaccharide (LPS) 10 ng/ml, R848 1 μg/ml, Poly(I:C) 25 μg/ml). Bar graph shows the frequency of IFNα^+^ cells in each subset with (+TLR) or without (NT) stimulation (*n* = 4 cord blood donors). *p < 0.05, one-way ANOVA test with Holm–Sidak’s multiple comparisons. Data are presented as floating bars ranging from min to max and line represents median (**a**, **b**, **d**).

We also observed that MS5_FS12 stromal cells significantly improve the differentiation of CD123^+^CD303/4^+^ cells (Fig. 3a), a phenotype shared by both plasmacytoid DC and pre/AS-DC^12,20^. A more refined phenotypic characterization of the in vitro-generated cells also shows that all CD123^+^ cells express high levels of CD45RA and they can be subdivided in AXL^−^CD327^lo/−^ and AXL^+^CD327^+^ subsets, phenotypically aligning to pDC and pre/AS-DC (Fig. 3b). This conclusion was further supported by gene set enrichment analysis (GSEA)^58^ of RNA-sequencing (RNA-seq) data, displaying a significant enrichment of previously reported pDC and AS-DC gene signatures^20^ in in vitro-generated AXL^−^CD327^lo/−^ and AXL^+^CD327^+^, respectively (Fig. 3c). Moreover, only the AXL^−^CD327^lo/−^ cells were capable to produce type I interferon in response to toll-like receptor (TLR) stimulation, a specific feature of bona fide pDC, which is not shared with pre/AS-DC^12,20^ (Fig. 3d).

In conclusion, we identified the combination of membrane-bound FLT3L, SCF, and CXCL12 (MS5_FS12) as the most efficient tested condition to support human DCs differentiation in vitro from CD34^+^ HSPCs.

### Human DC generated in vitro align with circulating blood DC

**Fig. 4:**
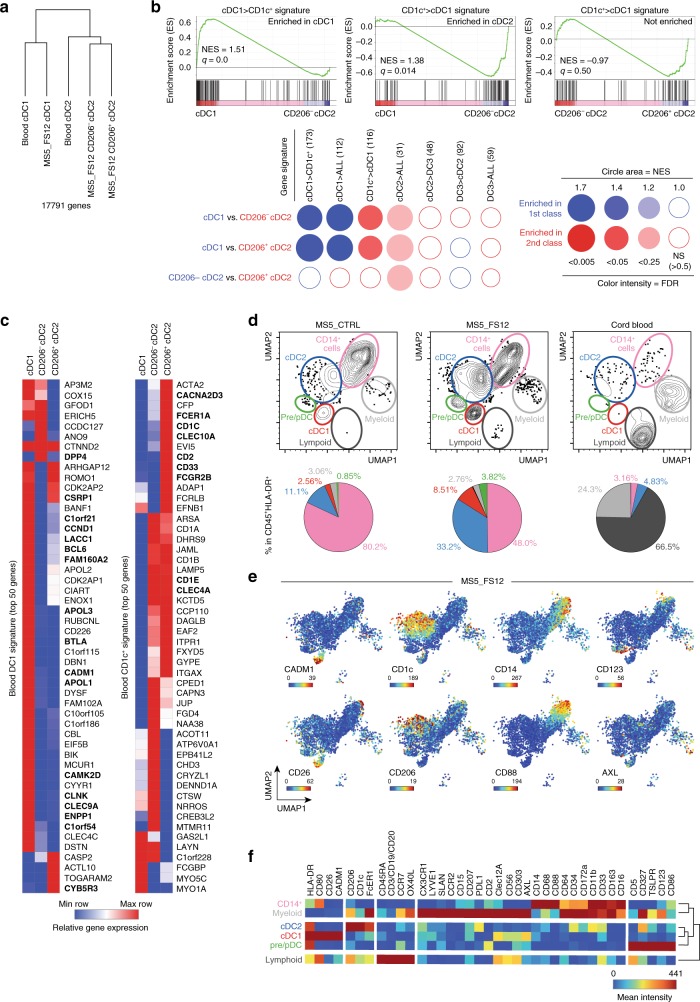
Human DC generated in vitro align with circulating blood DC. **a** Hierarchical clustering of primary (*n* = 3 healthy donors) vs. in vitro-generated (*n* = 3 cord blood donors) cDCs based on 17,791 genes after removing the “in vitro culture signature” (2000 genes) defined by pairwise comparison of primary versus in vitro generated subsets. **b** GSEA using blood cDC1s (DC1>CD1c^+^) and CD1c^+^ cells (CD1c^+^>DC1) signatures generated from published datasets^60^, as well as previously published signatures of blood cDC1 (DC1>ALL), cDC2 (DC2>ALL), and cDC3 (DC3>ALL)^20^. BubbleMap shows the enrichment of each gene signature in the pairwise comparison of CD141^+^Clec9A^+^, CD1c^+^CD206^−^, and CD1c^+^CD206^+^ cells generated in vitro (FDR false detection rate, NES normalized enrichment score). For single pairwise comparisons (top), statistical significance is defined by the FDR *q*-value calculated by the GSEA software (www.broad.mit.edu/gsea) using default parameters. For multiple pairwise comparisons (bottom), the statistical significance was further corrected for multiple testing by the BubbleMap module of BubbleGUM software. **c** Heatmaps of RNA-seq data displaying the expression of the top 50 genes of blood cDC1 and CD1c^+^ cells signatures in CD141^+^Clec9A^+^, CD1c^+^CD206^−^, and CD1c^+^CD206^+^ cells generated in vitro. Genes shared with previously published signatures^20^ are highlighted in bold. **d** UMAP (Uniform Manifold Approximation and Projection) plots of CyTOF data from CD45^+^HLA-DR^+^ cells differentiated in vitro using MS5_FS12 and MS5^_^CTRL as compared with cord blood PBMCs. Pie charts indicate the frequency of each subset among the CD45^+^HLA-DR^+^ cells (mean of *n* = 2 cord blood donors in two independent experiments). **e** Relative expression of selected markers in UMAP plots of CyTOF data from cells differentiated in vitro with MS5_FS12. **f** Heatmap of markers mean intensity in each subset identified in MS5_FS12 cultures.

To validate the identity of the cDCs generated using the MS5_FS12 stromal niche, we compared the transcriptome (RNA-seq) and phenotype (CyTOF) of in vitro-differentiated subsets to circulating blood cDC1s and cDC2s (Fig. 4a–f).

Hierarchical clustering of RNA-seq data revealed that subsets generated in culture maintain a strong “culture imprinting” (Supplementary Fig. 4a). Indeed, we could identify a 2000 genes signature (1000 genes up- and 1000 genes downregulated), which clearly separates in vitro-generated cells from circulating blood subsets regardless of their subset identity (Supplementary Fig. 4b). The majority of these genes were associated to cell cycle and metabolism as shown by pathways enrichment analysis (Supplementary Fig. 4c). Nonetheless, once this “in vitro culture signature” was subtracted from the total protein coding genes, CD141^+^Clec9A^+^ and CD1c^+^CD206^+/−^ cells generated in culture transcriptionally align to blood cDC1 and cDC2, respectively (Fig. 4a).

To further validate the similarity of in vitro generated cells with physiologically circulating subsets, we performed GSEA^58^ using the BubbleGum software^59^. This methodology enables to score the enrichment of a signature in a pairwise comparison of two transcriptomes. We scored cDC1 alignment using gene signatures specific for blood cDC1 obtained from published datasets (cDC1>CD1c^[+ 60^ and DC1>ALL^20^). CD14^−^CD1c^+^ cells have recently been shown to contain two distinct subsets termed as cDC2 and cDC3^20^. Alignment of cultured cells was probed towards total CD1c^+^ cells (CD1c>cDC1), cDC2 (cDC2>ALL and cDC2>DC3), and DC3 signatures (DC3>cDC2 and DC3>ALL). We found that in vitro-generated CD141^+^Clec9A^+^ and CD1c^+^CD206^+/−^ cells are enriched in genes defining circulating blood cDC1 and cDC2, respectively (Fig. 4b). The expression of the top 50 genes for each signature in the differentiated subsets further supports this conclusion (Fig. 4c). Importantly, both CD206^+^ and CD206^−^ subsets aligned preferentially to cDC2 as compared with DC3 and cDC1 (Fig. 4b and Supplementary Fig. 4d). CD163 was recently described as a marker selectively expressed in blood cDC3 as compared with cDC2^20^. Supporting our previous conclusion, CD163 was neither expressed in CD1c^+^CD206^−^ nor in CD1c^+^CD206^+^ cells generated in vitro, whereas CD163^+^ cells were detected among CD14^+^ monocytes and CD14^+^CD16^+^ macrophages (Supplementary Fig. 4e).

To obtain a more exhaustive characterization of the phenotype of in vitro-generated subsets, we performed CyTOF analysis using a panel of 38 metal-conjugated monoclonal antibodies. Dimension reduction of the CyTOF data was performed using the Uniform Manifold Approximation and Projection (UMAP) algorithm^61^. UMAP plots display clusters of cells that were expanded upon MS5_FS12 co-culture as compare to MS5_CTRL (Fig. 4d). Clec9A^+^CD141^+^ cells identified by flow cytometry were shown to also express CADM1 and CD26 further aligning them with blood cDC1s (red cluster, Fig. 4d–f). CD14^−^CD1c^+^ cells did not express high level of monocyte markers such as CD64, CD68, and CD16, whereas they appeared heterogeneous for CD206 expression (blue cluster, Fig. 4d–f and Supplementary Fig. 4f). Of note, CD14^−^CD1c^+^ cells generated in culture did not express high level of FcεRIa, CD172a, and CD5 found in blood cDC2s (Supplementary Fig. 4f). By contrast, they were strongly positive for CD86 and CD80 unlike their circulating counterpart (Supplementary Fig. 4f). In addition, CD123^+^CD303^+^ cells were shown to express heterogeneous levels of pre-DC markers such as CD327 and CX3CR1, and moderate level of AXL (green cluster, Fig. 4d–f and Supplementary Fig. 4f), in line with flow cytometry analysis highlighting the presence of both pDC and pre/AS-DC within CD123^+^CD303^+^ cells generated in vitro (Fig. 3b). On the other hand, by combining flow and mass cytometry analysis, we were able to show that MS5_FS12 stromal cells do not support lymphoid development (Fig. 4d and Supplementary Fig. 4g). Indeed, the remaining cells (other than DC) present in culture consist of CD15^+^ Granulocytes and CD14^+^CD16^+/−^ Monocytes/Macrophages (Supplementary Fig. 4h). Finally, the analysis of in vitro cDC differentiation kinetics revealed that both cDC1 and cDC2 can be detected in MS5_FS12 cultures as early as day7 (Supplementary Fig. 4i). However, the yield of in vitro generated cDC was significantly higher at day14, when most of the cultures were therefore analyzed (Supplementary Fig. 4i).

Collectively, our data demonstrate that: (i) in vitro-generated CD141^+^Clec9A^+^ recapitulate the phenotype of bona fide blood cDC1; (ii*)* CD14^−^CD1c^+^ cells align to cDC2 regardless of their CD206 expression; (iii*)* CD123^+^CD303^+^ cells contain some recently described pre-DC/AS-DC phenotypically and functionally distinct from pDCs. However, we identified two major limitations of the in vitro culture. First, the culture system imposes a strong transcriptional imprinting throughout subsets. Second, in vitro-generated cDC2s failed to express to full phenotypic profile of blood cDC2s.

### Engineered stromal niches support HSPC maintenance in vivo

We next wanted to assess whether we could use MS5_FS12 to recapitulate a more physiological niche microenvironment supporting human HSPCs maintenance in vivo.

**Fig. 5:**
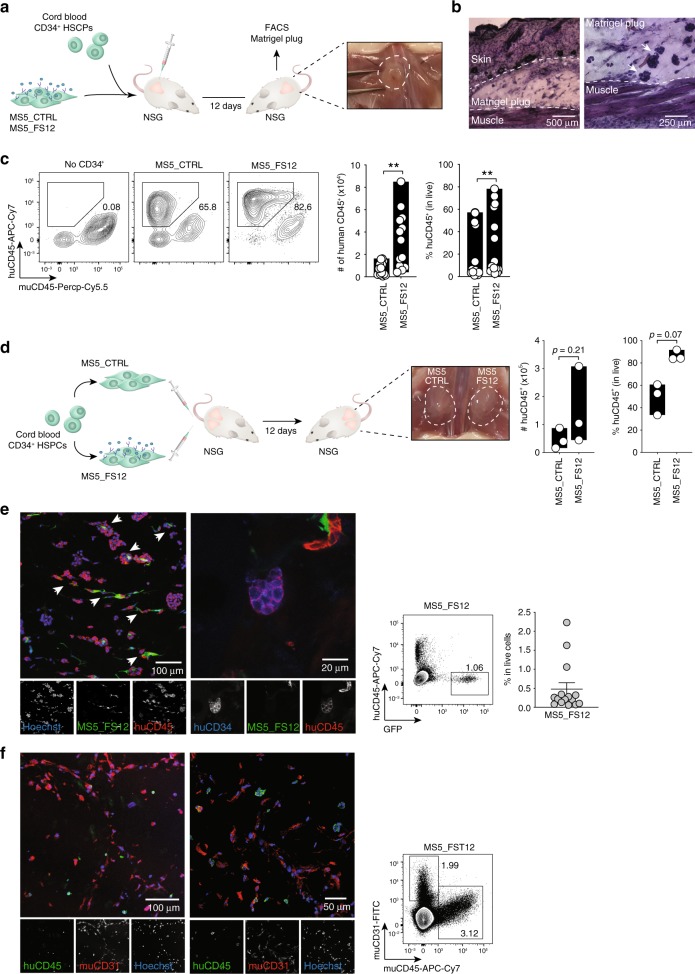
Engineered stromal niches support HSPC maintenance in vivo. **a** Experimental strategy for an in vivo synthetic niche. Human HSPCs were injected subcutaneously along with MS5_FS12 in a basement membrane matrix (Matrigel) preparation. **b** Hematoxylin–eosin staining of subcutaneous organoids at day 12. Arrows show clusters of Matrigel-embedded cells. Scale bar represents 500 μm (left) and 250 μm (right). **c** Flow cytometry analysis at day 12 of Matrigel organoids containing either MS5_CTRL or MS5_FS12 cells. Absolute number and frequency of human CD45^+^ cells recovered are summarized in bar graphs (*n* = 13 cord blood donors in 6 independent experiments; **p < 0.01, two-tailed paired Student’s *t*-test). **d** Experimental strategy and quantification of human CD45^+^ cells recovered from physically separated plugs containing either MS5_CTRL or MS5_FS12 cells injected in the same recipient (*n* = 3 cord blood donors in one experiment; two-tailed paired Student’s *t*-test). **e** Immunofluorescence staining of plug sections displaying the interaction of GFP^+^ MS5_FS12 (green) with human CD45^+^ cells (red). Human hematopoietic progenitors were also identified as CD45^+^ (red) CD34^+^ (blue) cells in MS5_FS12 plugs. Nuclei were stained with Hoechst (blue). Arrows show interaction of human CD45^+^ leukocytes with GFP^+^ MS5_FS12. Scale bar represents 100 μm (left panel) and 20 μm (right panel). Similar results were observed in *n* = 5 Matrigel organoids. The presence of GFP^+^ stromal cells in Matrigel organoids at day 12 was further confirmed by flow cytometry (*n* = 15 independent organoids). **f** Visualization of mouse CD31^+^ endothelial cells by immunofluorescence. Fixed sections were stained for human CD45 (green) and mouse CD31 (red). Nuclei were stained with Hoechst (blue). Scale bar represents 100 μm (left panel) and 50 μm (right panel). Similar results were observed in *n* = 5 Matrigel organoids. The presence of mouse CD31^+^ cells was further confirmed by flow cytomery. Data are presented as floating bars ranging from min to max and line represents median (**c**, **d**) or s**c**atter plots with mean ± SEM (**e**).

To this end, we designed an experimental strategy based on the subcutaneous injection of cord blood-derived CD34^+^ HSPCs together with MS5_FS12 in a basement membrane matrix (Matrigel) in NOD.Cg-*Prdc*^*scid*^ *Il2rg*^*tm1Wjl*^*/SzJ* (NSG) mice (Fig. 5a).

Clusters of cells embedded in Matrigel can be identified as early as day 12 by tissue histology (Fig. 5b). Flow cytometry analysis demonstrated that MS5_FS12 but not MS5_CTRL induced the expansion of human leukocytes within the Matrigel plugs (Fig. 5c). We then tested whether cell-to-cell interactions of eMSC with human progenitors play a role in this process. We injected two independent plugs of CD34^+^ HSPCs with either MS5_CTRL or MS5_FS12 in the same recipient mouse (contralateral plugs) (Fig. 5d). We found a relative expansion of human leukocytes in MS5_FS12 as compared to MS5_CTRL contralateral plugs (Fig. 5d). We conclude that MS5_FS12 does not efficiently provide soluble factors enabling human leukocytes expansion systemically. Therefore, we hypothesized that membrane-bound FLT3L and SCF together with CXCL12 define an efficient in vivo niche by delivering cell-to-cell contacts supporting HSPCs expansion. Supporting this hypothesis, we found that MS5_FS12 expressing GFP persist in the plugs at day 12 of differentiation (Fig. 5e). Immunofluorescence analysis further supported this observation and demonstrated the existence of cell-to-cell contact between MS5_FS12 and human CD45^+^ leukocytes (Fig. 5e and Supplementary Fig. 5a). Leukocytes expressing CD34^+^ could also be detected, supporting the notion that a pool of undifferentiated progenitors is maintained in the MS5_FS12 organoids at day 12 (Fig. 5e). Of note, Matrigel plugs contained mouse CD31^+^ cells, suggesting undergoing vascularization as evidenced by the formation of early tube-like structure (Fig. 5f). However, no vascular leak was observed, as demonstrated by the absence of intravenously delivered CTV^+^CD3^+^ cells in the subcutaneous plug (Supplementary Fig. 5b).

Taken together, these data show that engineered stromal cells MS5_FS12 provide a minimal synthetic niche scaffold supporting human CD34^+^ HSPCs maintenance and expansion in vivo.

### The MS5_FS12 niche supports human DC development in vivo

We investigated whether the engraftment of CD34^+^ HSPCs together with MS5_FS12 could lead to the local differentiation of human DC subsets.

**Fig. 6:**
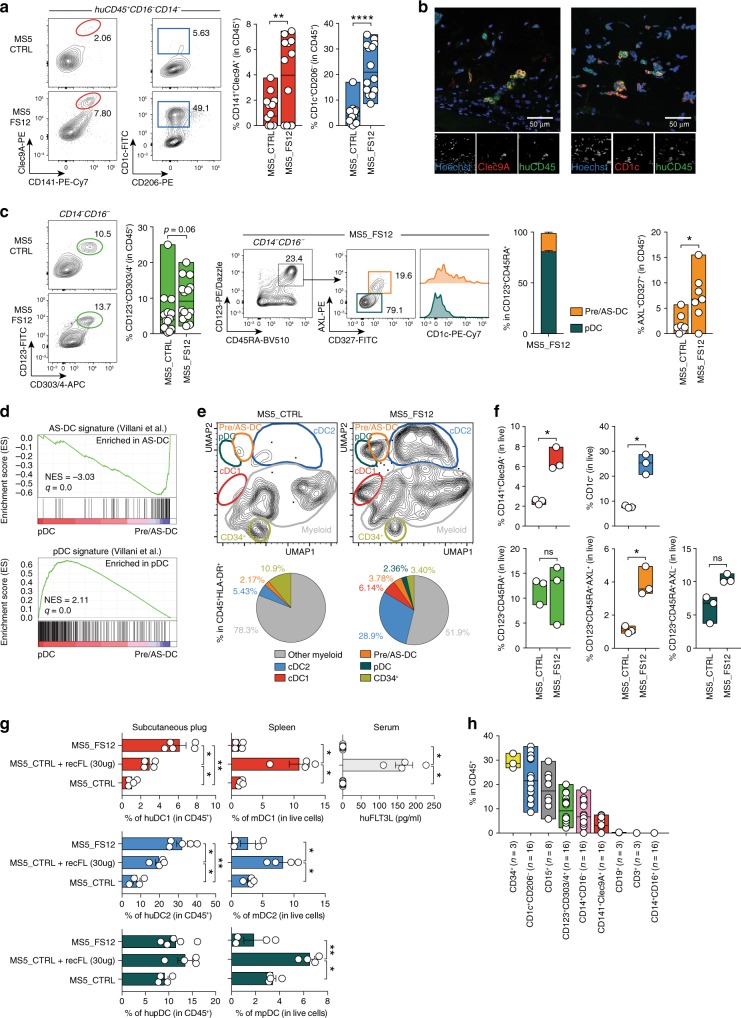
The MS5_FS12 niche supports human DC development in vivo. **a** Flow cytometry of Matrigel organoids containing either MS5_CTRL or MS5_FS12. Graphs show frequency of cDC1 and cDC2 within CD45^+^ cells (*n* = 14 donors in 6 independent experiments). **p < 0.01 ****p < 0.0001, two-tailed paired Student’s *t*-test. **b** Immunofluorescence of MS5_FS12 organoids sections stained for huCD45^+^ (green) and Clec9A^+^ (red) or and CD1c^+^ (red). Nuclei stained with Hoechst (blue). Scale bar = 50 μm (*n* = 2 Matrigel organoids). **c** Left: Flow cytometry and quantification of CD123^+^CD303/4^+^ cells in MS5^_^CTRL and MS5_FS12 organoids (*n* = 14 donors in 6 independent experiments. Two-tailed paired Student’s *t*-test). Middle: Gating strategy discriminating AXL^−^CD327^lo/−^ pDC and AXL^+^CD327^+^ pre/AS-DC within CD123^+^CD45RA^+^ cells in MS5^_^FS12 organoids. Bar graph illustrates frequency of each subset within CD123^+^CD45RA^+^ cells (*n* = 4 donors). Right: Frequency of pre/AS-DC within CD45^+^ cells in MS5_CTRL vs. MS5_FS12 organoids (*n* = 7 donors in 4 independent experiments). *p < 0.05, two-tailed paired Student’s *t*-test. **d** GSEA of pDC and pre/AS-DC using published gene signatures^20^. Statistical significance defined by the FDR *q*-value calculated by GSEA software (www.broad.mit.edu/gsea) using default parameters. **e** CyTOF analysis comparing CD45^**+**^HLA-DR^+^ cells in MS5_FS12 and MS5^_^CTRL organoids. Pie charts display frequency within CD45^+^HLA-DR^+^ cells (mean of *n* = 2 donors in 2 independent experiments). **f** Frequency of cDC1, cDC2, pDC, pre/AS-DC and total CD123^+^CD45RA^+^ cells recovered from physically separated plugs containing either MS5_CTRL or MS5_FS12 in the same recipient (*n* = 3 donors in one experiment). *p < 0.05, two-tailed paired Student’s *t*-test. **g** NSG mice injected subcutaneously either with MS5_CTRL or MS5_FS12 stromal cells. Human recombinant FLT3L administered intra-peritoneum to mice bearing MS5_CTRL plugs (10 μg/mouse/injection) (MS5_CTRL+recFL). Frequency of human cDC1, cDC2, and pDC in subcutaneous organoids (left) and murine cDC1, cDC2 and pDC in the spleen (center) were reported. Circulating recombinant FLT3L levels were measured by ELISA (right). *n* = 4 mice/group in 2 independent experiments. *p < 0.05, **p < 0.01, one-way ANOVA test with Dunnett’s T3 multiple comparisons. **h** Frequency of differentiated subsets within huCD45^+^ cells in MS5**_**FS12 plugs at day 12. The number of biological replicates (*n*) is reported. Data presented as floating bars ranging from min to max and line represents median (**a**, **c**, **f**, **h**) or as bar graphs with mean ± SEM (**c**, **g**).

Flow cytometry analysis of Matrigel organoids demonstrates that the MS5_FS12 but not the MS5_CTRL niche specifically supports the differentiation of CD141^+^Clec9A^+^ cDC1-like cells and CD14^−^CD1c^+^ cDC2-like cells (Fig. 6a and Supplementary Fig. 6a). This finding was supported by immunofluorescence staining on plug sections highlighting the occurrence of human CD45 cells expressing either Clec9A or CD1c (Fig. 6b).

Further analysis revealed the expansion of CD123^+^CD303/4^+^ cells in MS5_FS12 when compared to MS5_CTRL plugs (Fig. 6c and Supplementary Fig. 6a). All these cells also expressed CD45RA and heterogeneous levels of AXL and CD327, as previously described for their in vitro counterparts (Fig. 6c). However, only MS5_FS12 induced a strong accumulation of AXL^+^CD327^+^ pre/AS-DC expressing various levels of CD1c (Fig. 6c). In addition, bona fide CD123^+^CD45RA^+^AXL^−^CD327^lo/-^ pDCs could also be detected (Fig. 6c). RNA-seq analysis of in vivo-generated CD123^+^AXL^−^CD327^lo/−^ and CD123^+^AXL^+^CD327^+^ cells further support this conclusion and unequivocally align them to blood circulating pDC and AS-DC, respectively (Fig. 6d).

To further refine the phenotypic characterization of HLA-DR^+^ mononuclear phagocytes in MS5_FS12 organoids we performed high-dimensional mass cytometry analysis. The comparison of MS5_FS12 with MS5_CTRL plugs highlighted the expansion of all subsets previously identified by flow cytometry: cDC1s, cDC2s, pre/AS-DCs, pDCs, and a distinct population of CD33^+^CCR2^+^CX3CR1^+^Clec12A^+^ myeloid cells (Fig. 6e and Supplementary Fig. 6b). We next wanted to determine whether commitment towards the cDC lineage would be dependent on local developmental cues and possibly cell-to-cell contact between CD34^+^ HSPCs and MS5_FS12. To this end, we engrafted mice with two distal organoids, one containing MS5_CTRL and the second one containing MS5_FS12. We found that cDC1, cDC2, and pre/AS-DCs were selectively expanded in MS5_FS12 plugs (Fig. 6f and Supplementary Fig. 6c). On the contrary, pDC were not significantly increased in the same comparison (Fig. 6f and Supplementary Fig. 6c). We conclude that local cues associated to the MS5_FS12 niche control cDC lineage commitment. In support of this view, we could not detect a systemic increase in the levels of serum FLT3L in mice carrying engineered stromal cell plugs (Fig. 6g). Accordingly, spleen resident murine cDCs did not expand upon MS5_FS12 engraftment, while they massively do so upon administration of recombinant soluble human FLT3L (Fig. 6g). Together with the two-plugs experiments (Fig. 6f), these observations suggest that most of the FLT3L aegis relies on its membrane-bound form delivered in the context of eMSCs. Of note, administration of recombinant soluble FLT3L was poorly efficient at expanding human DCs populations in Matrigel organoids formed with control stromal cells (Fig. 6g). This demonstrates the superiority of local, cell-associated cues (i.e., MS5_FS12) to achieve the expansion of human cDCs in the dermis of NSG mice.

A more extensive characterization of MS5_FS12 organoids revealed the presence of myeloid lineages other than DCs, such as CD14^+^CD16^−^ monocyte-like cells and CD15^+^ granulocytes (Fig. 6h and Supplementary Fig. 6d). Conversely, no lymphoid specification was observed (Fig. 6h and Supplementary Fig. 6d). Despite this broad spectrum of lineages, CD34^+^ HSPCs represented the most abundant population at day 12 (Fig. 6h). This observation suggests that the MS5_FS12 niche combines HSPC maintenance with lineage commitment.

### cDC2 generated in vivo faithfully align to blood cDC2

Finally, we wanted to establish whether in vivo differentiated DCs in MS5_FS12 organoids had a distinct phenotype from the subsets generated in vitro in MS5_FS12 co-culture.

**Fig. 7:**
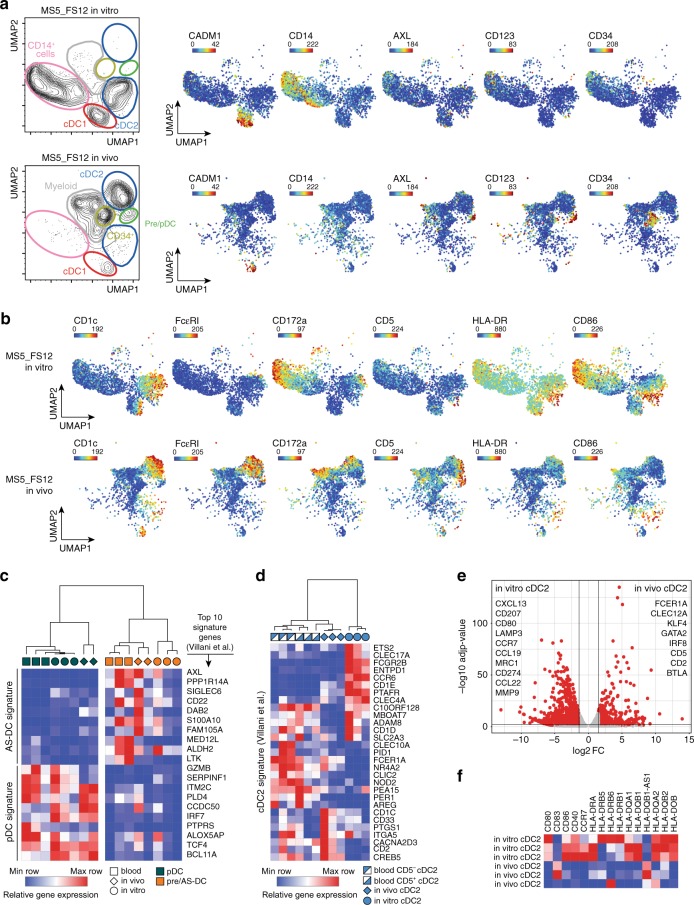
cDC2 generated in vivo faithfully align to blood cDC2. **a** UMAP plots of CyTOF data comparing CD45^+^HLA-DR^+^ cells generated using MS5_FS12 stromal cells either in vitro or in vivo. Relative expression of selected markers is shown for each condition. **b** Relative expression of selected markers highlighting the phenotypic differences between cDC2s generated in vitro and in vivo using MS5_FS12 stromal cells. **c** Heatmap displaying gene expression of the top ten genes of blood pDC and AS-DC published signatures^20^ in pDC and pre/AS-DC generated in vitro, in vivo, and isolated from blood PBMC (*n* = 2 independent donors for pDC and pre/AS-DC generated in vivo and *n* = 3 independent donors for pDC and pre/AS-DC generated in vitro or isolated from peripheral blood). **d** Heatmap displaying gene expression of the blood cDC2 published signature^20^ in cDC2 cells generated in vitro, in vivo and isolated from blood PBMC (*n* = 3 independent donors). **e** Volcano plot showing differentially expressed genes between in vitro and in vivo generated cDC2 (Log2FC > 1.5, adjusted *p*-value < 0.05). Statistical significance was calculated using Wald test with a Benjamin–Hochberg *p*-value correction (*n* = 3 independent donors per group). **f** Heatmap displaying gene expression of activation markers and co-stimulatory molecules expressed in cDC2 generated in vivo and in vitro (*n* = 3 independent donors).

UMAP plots of CyTOF analysis revealed three major findings. First, pre/AS-DCs represent a more abundant population in vivo (Fig. 7a and Supplementary Fig. 7a). Second, both cDC1 generated in vitro and in vivo fully align phenotypically, displaying a strong expression of CADM1 and CD26 (Fig. 7a and Supplementary Fig. 7a). Third, unlike cDC1, cDC2 generated in vivo exhibit noticeable phenotypic differences. In vivo-generated cDC2 express higher levels of FcεRIa, CD172a, and CD5, while showing lower expression of HLA-DR and CD86 (Fig. 7b and Supplementary Fig. 7b). The specific phenotype conferred by the MS5_FS12 niche education renders cDC2s more akin to their blood counterparts. In order to compare extensively the transcriptional landscape of in vivo (NSG organoids) generated DCs with primary DCs found in human blood, we performed RNA-seq analysis on fluorescence-activated cell sorting (FACS)-sorted cDC2s obtained after the enzymatic digestion of MS5_FS12-containing plugs or purified from human blood.

As previously observed for in vitro-generated cells, the MS5_FS12 niche confers an in vivo imprinting resulting in the differential expression of 2872 genes (up- or downregulated) in in vivo versus ex vivo-isolated subsets (Supplementary Fig. 7c). Pathway analysis revealed that this in vivo bias was mainly due to upregulation of genes associated with DNA replication, cell cycle and proliferation (*MYC*, *CDC6/7*, *POLA2*, *MCM6/7*) (Supplementary Fig. 7c and Supplementary Table 8).

Moreover, we found that: (i) AXL^+^Siglec6^+^ pre/AS-DCs generated in vivo (or in vitro) align to their primary counterparts and selectively express a signature that distinguish them from bona fide pDCs (*DAB2*, *CD22*, *ADLH2*) (Fig. 7c). (ii) conversely, AXL^−^Siglec6^−^ bona fide pDCs generated in vivo (or in vitro) align to their primary counterparts and express high levels of markers distinguishing them from pre/AS-DCs (*IRF7*, *GZMB*, *TCF4*, *BCL11A*) (Fig. 7c). (iii) cDC2s generated in vivo (in NSG mice organoids carrying MS5_FS12) had higher levels of similarity with blood cDC2s (including higher expression of *BTLA*, *FCER1A*) (Figs. 7b, d, e). Recently, both CD5^+^ and CD5^−^ cDC2s subsets have been reported in human blood^62,63^ and we found that in vivo generated cDC2s aligned particularly well with blood CD5^+^ cDC2s (with the expression of *CD5*, *CD2*) (Figs. 7b, e). By contrast, in vitro-generated cDC2s expressed high levels of activation genes such MHC molecules (*HLA-DR*, *DQ*), co-stimulatory molecules (*CD80, CD40*), activation markers (*ETS2, CCR6, CCR7, CXCL13, CCL22*) (Figs. 7e, f) and genes associated with type I and type II interferon pathways (*STAT1*, *IRF9*, *IGS15*, *GBP1*) (Supplementary Fig. 7d and Supplementary Table 9).

All together, we conclude that MS5_FS12-containing organoids provide a unique scaffold for the specification and commitment of the DC lineage. This unique and versatile system bypasses the limitation of in vitro cultures, which generated inefficiently pre/AS-DCs and biased the differentiation of cDC2s toward an activated phenotype. Collectively, MS5_FS12 organoids faithfully recapitulate the differentiation of not only pDCs, cDC1s, and cDC2s but also pre/AS-DCs, and support the development of cDC2s displaying an unattained level of similarity with their human blood counterparts.

### In vitro and in vivo cDC2 functionally align to blood cDC2

In the last set of experiments, we aimed at functionally validate cord blood-derived cDC generated in the MS5_FS12 stromal niche. Moreover, we also assessed whether the phenotypic differences observed in cDC2 generated in vitro and in vivo may impact their function.

**Fig. 8:**
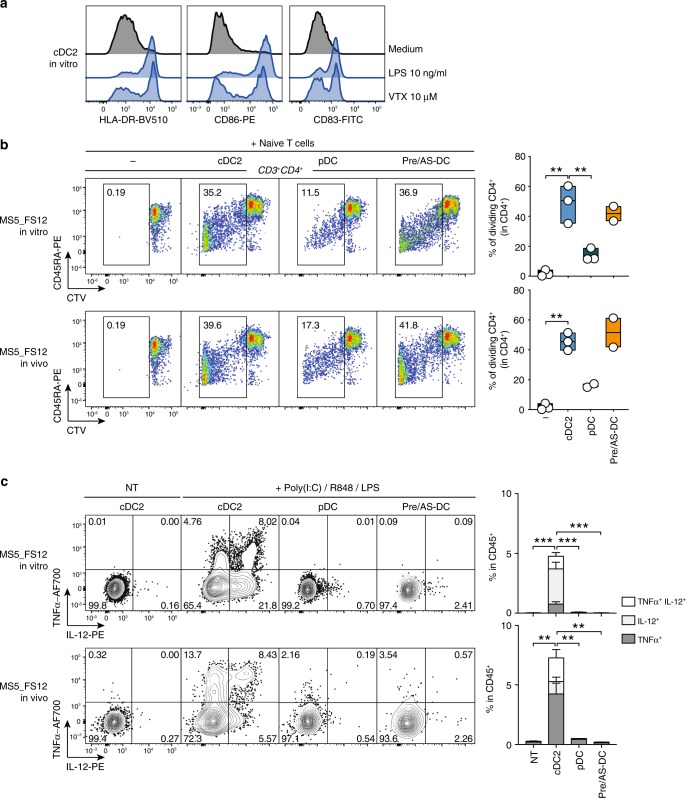
In vitro and in vivo cDC2 functionally align to blood cDC2. **a** Histograms showing upregulation of activation markers HLA-DR, CD86 and CD83 in in vitro-differentiated cDC2 in response to TLR4 (LPS) and TLR8 (VTX-2337) overnight (16 h) stimulation. **b** Representative FACS plots and quantification of mixed lymphocyte reaction (MLR) using in vitro and in vivo differentiated cDC2, pDC, and pre/AS-DC. FACS-sorted DC subsets were activated overnight (16 h) using a TLR agonist cocktail (LPS 10 ng/ml, R848 1 μg/ml and Poly(I:C) 25 μg/ml) and co-cultured with CTV-labeled naive T cells for 7 days (*n* = 2 independent donors for pDC generated in vivo and pre/AS-DC generated in vitro and in vivo and *n* = 3 independent donors for in vitro and in vivo generated cDC2 and in vitro generated pDC in two independent experiments). **p < 0.01, one-way ANOVA test with Holm–Sidak’s multiple comparisons. **c** Intracellular flow cytometry analysis of TNFα and IL-12 expression in in vitro and in vivo-differentiated DC subsets upon overnight (16 h) stimulation with TLR agonist cocktail (LPS 10 ng/ml, R848 1 μg/ml, and Poly(I:C) 25μg/ml) as compared to unstimulated cells (NT). *n* = 4 independent cord blood donors. **p < 0.01, ***p < 0.001, one-way ANOVA test Holm–Sidak’s multiple comparisons. Data are presented as floating bars ranging from min to max and line represents median **b** or as bar graphs with mean ± SEM (**c**).

We first confirmed in vitro the responsiveness of cord blood-derived cDC2 to TLR agonists expressed in human circulating cDC2, as demonstrated by the upregulation of maturation markers (i.e., HLA-DR, CD86, and CD83) in response to TLR4 (LPS) and TLR8 (VTX-2337) stimulation (Fig. 8a). We then performed a mixed lymphocyte reaction (MLR) by co-culturing CTV-labeled allogeneic naive T cells together with FACS-sorted cDC subsets (Supplementary Fig. 8a) activated overnight with a TLR agonists cocktail comprising of LPS (TLR4), R848 (TLR7/8) and Poly(I:C) (TLR3). After 7 days of culture, we observed that both in vitro and in vivo-generated cDC2 and pre/AS-DC were capable to efficiently induce CD4^+^ naive T-cell proliferation (Fig. 8b), as expected and reported for circulating blood cDC2^12,20^ (Supplementary Fig. 8b). Conversely, pDC were significantly less effective on triggering T-cell activation, as shown by the consistent reduction in the frequency of dividing CD4^+^ T cells when compared with cDC2 and pre/AS-DC (Fig. 8b).

Importantly, only cDC2 were able to produce high amounts of T-cell-polarizing cytokines in response to TLR stimulation, as demonstrated by intracellular TNFα and IL-12 detection by flow cytometry (Fig. 8c). All these features demonstrate that cDC2s, pDCs and pre/AS-DCs functionally align to their in vivo counterparts as previously described in the literature^12,20^.

Collectively, our data suggest that: (i) both in vitro and in vivo differentiated cDC2 are equally capable to induce CD4^+^ T cells activation and produce large amounts of TNF-α and IL-12; (ii) pre/AS-DC are as efficient as cDC2 in activating allogeneic naive CD4+ T cells in vitro, a distinctive feature that clearly separate them from the pDC lineage; (iii) despite their ability to induce CD4^+^ T-cell proliferation in MLR settings, pre/AS-DC do not produce high levels of cytokines commonly associated with cDC2 function, such as TNF-α or IL-12.

## Discussion

Over the last two decades, DC-based strategies have been proposed for the therapeutic vaccination against cancer, including (i) non-targeted protein-based vaccines captured by DCs in vivo, (ii) specific targeting of DC subsets with mAb coupled to tumor antigens^64^ and (iii) antigen loading of ex-vivo-generated DCs^3^. In this context, experimental models recapitulating the development of human DC subsets are crucially needed.

Here we describe a novel approach to model human DC development from CD34^+^ HSPCs both in vitro and in vivo. To this end, we primarily focused on the physiological niches where human DCs differentiate and maintain: a central bone marrow niche where DC progenitors are specified and peripheral niches in the lymph nodes where DCs reside.

Previous studies have shown that the cell-to-cell interaction with membrane-bound factors expressed by the niche microenvironment plays an essential role in HSPCs maintenance and expansion^21,22,65,66^. Alternative splicing of human and murine SCF transcript results in the synthesis of both a soluble and a membrane-bound non-cleavable form of the protein. Interestingly, the secreted form of SCF/KITLG is not sufficient for the establishment of a functional niche in murine bone marrow^21,22^, whereas the expression of human membrane-bound SCF is sufficient to support human myeloid development in humanized mice^66^. We therefore wanted to test whether a similar relationship might exist between the soluble and membrane-bound forms of human FLT3L. Consistent with this hypothesis, the expression of transmembrane FLT3L in mesenchymal stromal cells (MS5_F) improved the efficiency of DC differentiation in vitro, as compared to its soluble form (MS5 + recFL). Moreover, the engraftment of distal organoids (MS5_CTRL vs. MS5_FS12) together with the comparison of local (membrane-bound) vs. systemic (soluble) delivery of human FLT3L in vivo, supported the notion that cell-associated FLT3L delivered by engineered stromal cells significantly improves the development of human DCs in NSG mice.

Several protocols have been proposed for the in vitro differentiation of human cDCs from CD34^+^ HSPCs^7,37–39,46,47^. In vitro-differentiated cDC1s have been shown to fully recapitulate both the phenotype and function of circulating bona fide blood cDC1s^8,38,46,47^ including high levels of IRF8 expression and Batf3-dependency in vitro^13^, as well as IRF8-dependancy both in vivo^67^ and in vitro^46^. Conversely, several aspects have limited an exhaustive validation of in vitro generated CD1c^+^ cDC2-like cells such as the expression of CD14^7^, the transcriptional alignment with monocyte-derived DCs^38^ or the lack of a high-dimensional phenotypic characterization^46,47^.

Here we described a system (MS5_FS12), which efficiently supports the differentiation of both CD141^+^Clec9A^+^ cDC1s and CD14^−^CD1c^+^ cDC2s. However, cDC2s generated in MS5_FS12 cultures only partially recapitulate the phenotype of circulating blood cDC2s, as suggested by the lack of expression of cDC2-specific markers such as FcεRIa and CD5. Nevertheless, engineered MSCs display the unique advantage of being suitable for in vivo applications.

Immunodeficient mice provide a unique system to model the onset of human immune responses in realistic settings^68^. However, a reliable method to achieve the differentiation of human DCs in vivo has not been described, yet. Current protocols rely on the engraftment of human CD34^+^ HSPCs in sub-lethally irradiated immunodeficient mice (humanized mice). This strategy has not been successful in the generation of well-characterized circulating DC subsets^68^. Administration of supraphysiological levels of recombinant FLT3L has been shown to stimulate cDC differentiation upon reconstitution of NSG^28,48^ or *Flt3*^−/−^ BRGS^49^ mice with human CD34^+^ HSPCs. However, the phenotype of CD141^+^ cDC1s and CD1c^+^ cDC2s was poorly characterized and, despite exceptions^50^, tissue DCs were not generally investigated. These aspects represent an important limitation by precluding, for instance, the modeling of skin vaccination. Alternatively, transgenic mice expressing human GM-CSF and IL-3 (in the presence or absence of human SCF), either constitutively^69,70^ or by replacing their murine counterparts (knock-in)^71^, have been generated. Despite displaying higher levels of myeloid reconstitution, as well as the presence of human alveolar macrophages in the lungs of humanized mice^71^, this approach did not improve the development of human cDCs in lymphoid and non-lymphoid tissues of engrafted animals.

We demonstrated that MS5_FS12 support the differentiation of human cDCs in vivo in subcutaneous organoids in NSG mice. High-dimensional mass cytometry (Cytof) and transcriptomic (RNA-seq) analysis of in vivo generated cells confirmed their phenotypic and transcriptional alignment to circulating blood cDC1s and cDC2s. More importantly, cDC2s generated in vivo better resemble their physiologically circulating counterparts by expressing higher levels of FcεRIa, CD172a, CD5, CD2, and BTLA when compared to in vitro differentiated cells. The lower expression of activation markers such as CD86, CD80 and MHC molecules also suggests that in vivo cDC2s displayed a less mature phenotype than in vitro-generated cells.

Moreover, MS5_FS12 niche was capable of supporting the local maintenance and expansion of human HSPCs as well as pre/AS-DCs, resulting in the persistence of a long-lasting source of progenitors capable of undergoing DC differentiation. To our knowledge, this is the first time that a well-characterized system supporting the development of human pre/AS-DC is reported.

Collectively, we have demonstrated that the engineered stromal cells MS5_FS12 give rise to a synthetic hematopoietic niche when injected subcutaneously in NSG mice. The niche microenvironment efficiently supports the expansion of CD34^+^ HSPCs and human DCs subsets (cDC1, cDC2, and pre/AS-DC) can be detected as early as day 12 in a radiation-free environment. Importantly, in vitro culture system imposes a certain level of spontaneous activation that is not found in primary circulating blood DCs. Differentiation of human cDCs within humanized mice limit this phenomenon to a level closer to the maturation state of circulating primary cDCs. Hence, this approach represents a versatile system to study human DC development and function in vivo.

## Methods

### Mice

All in vivo experiments were performed using NOD.Cg-*Prdc*^*scid*^ *Il2rg*^*tm1Wjl*^/SzJ (NSG) mice (JAX #005557). All mice were used between 8 and 12 weeks of age. They were maintained in specific-pathogen-free conditions and handled according to protocols approved by the UK Home Office.

### Generation of engineered MSCs

Human FLT3L, SCF, and TPO were amplified by PCR from cDNA expression plasmids (Origene) and cloned into pMX retroviral vectors (vectors details in Supplementary Table 1). Lentiviral vector pBABE-puro-SDF-1 alpha was a gift from Bob Weinberg (Addgene plasmid #12270)^72^. Viral particles were generated using the retroviral packaging plasmid pCL-Ampho and a second generation lentiviral packaging system (psPAX2 and pMD2.G), respectively. MS5 cells were first transduced with CXCL12 lentiviral vector and selected using 15 μg/ml of Puromycin (Thermo Fisher). Then, a combination of single or multiple cytokines were used to transduce MS5 cells as illustrated in Supplementary Fig. 2a. Cells expressing human membrane-bound FLT3L and SCF were sorted according to antibody staining of the transmembrane proteins (antibodies listed in Supplementary Table 3). TPO-expressing cells were sorted according to the expression of mCherry reporter.

### Flow cytometry and fluorescent-activated cell sorting

Extracellular staining of cells was preformed in FACS buffer, consisting in phosphate-buffered saline (PBS) (Life Technologies), 1% bovine serum albumin (BSA) (Apollo Scientific) and 2 mM EDTA (Life Technologies). For intracellular staining, samples were fixed and permeabilized using the Cytofix/Cytoperm^TM^ kit (BD Biosciences) according to manufacturers’ instructions. Antibodies used in all experiments are listed in Supplementary Table 3. Flow cytometry analysis was performed on LSR Fortessa II (BD Biosciences, BD FACSDiva Software) and data were analyzed using FlowJo software (TreeStar, version 10.2). Cell sorting was performed using AriaII (BD Biosciences, BD FACSDiva Software).

### Cell lines maintenance and primary cells isolation

MS5^51^ and eMSC lines were cultured in IMDM (Life Technologies) supplemented with 10% heat-inactivated fetal bovine serum (FBS) (Life Technologies), penicillin/streptomycin (Life Technologies), 50 μM β-mercaptoethanol (Life Technologies), and maintained at 37 °C 5% CO_2_. OP9 and OP9_FLT3L were cultured in complete α-MEM (Life Technologies) supplemented with 20% not heat-inactivated FBS, penicillin/streptomycin, 50 μM β-mercaptoethanol and maintained at 37 °C 5% CO_2_.

Cord blood units were obtained from Anthony Nolan Cell Therapy Centre (ANCTC). Peripheral blood mononuclear cells (PBMCs) were isolated by gradient centrifugation using Ficoll-Paque (GE Healthcare) and CD34^+^ hematopoietic progenitors were isolated using CD34 MicroBead Kit UltraPure (Miltenyi Biotec).

Adult peripheral blood was obtained from healthy volunteers from NHS Blood and Transplant. PBMCs were isolated by Ficoll-Paque gradient centrifugation. Cells were collected in FACS buffer and used for downstream applications.

### Human dendritic cell differentiation in vitro

For in vitro differentiation of human DCs, MS5, OP9 or eMSCs were seeded in a 96-well plate (flat bottom) at a density of 10^4^ cells/well. The following day, 10^4^ cord blood-derived CD34^+^ cells/well were seeded on top of stromal cells in complete IMDM (10% heat-inactivated FBS, penicillin/streptomycin, 50 μM β-mercaptoethanol) and maintained at 37 °C 5% CO2. Half of the medium was replaced at day 5 and 10, and cells were collected with a solution of PBS 5 mM EDTA (at 4 °C) at day 15 for flow cytometry analysis. For recombinant FLT3L experiments, human FLT3L (Celldex) 100 ng/ml was used. For transwell experiments, 24-well plates with 0.4 μm pores Transwell® inserts (Corning) were used. Stromal cells were plated at a density of 10^5^ cells/well and 7 × 10^4^ cord blood-derived CD34^+^ progenitors were added the following day in each well. Half of the medium was replaced in both the top and bottom well at day 5 and 10. For GM-CSF blocking experiments, 2 μg/ml human GM-CSF neutralizing antibody (R&D catalog number #AF-215-SP) and isotype control (R&D catalog number #AB-108-C) were added to the culture medium. The presence of human GM-CSF in the supernatant of eMSCs co-cultures with CD34^+^ HSPCs was assessed by enzyme-linked immunosorbent assay (ELISA) using the human GM-CSF ELISA MAX kit (Biolegend) as per manufacturer’s instructions.

### RNA-seq and data processing

RNA-seq analysis was performed in two independent experiments. In the first experiment, human cDC1s and cDC2s from peripheral blood of *n* = 3 healthy individuals and in vitro generated CD141^+^Clec9A^+^, CD14^-^CD1c^+^CD206^−^, and CD14^−^CD1c^+^CD206^+^ cells from *n* = 3 independent cord blood donors were FACS sorted. Up to 100 cells/subset were collected in lysis buffer (Takara Clontech) containing RNAse inhibitors. RNA-seq libraries were prepared using Labcyte Echo 525 contactless liquid handling system (Labcyte, Inc.). In brief, ERCC mix (Thermo Fisher) was added to each sample and first strand full-length cDNA was generated with a modified protocol of the SMARTseq v4 Ultra Low Input RNA Kit (Takara Clontech) using poly dT primers and a template switching oligo. Full-length cDNA was amplified using SeqAmp™ DNA Polymerase (Takara Clontech). 12 ng of amplified cDNA from each sample was used to generate non-stranded RNA libraries using a modified protocol of the Ovation Ultralow System V2 1-96 kit (NuGEN). In brief, amplified cDNA was fragmented through sonication on Covaris E220 (Covaris, Inc.), repaired and polished followed by ligation of indexed adapters. Adapter ligated cDNA were pooled before final amplification to add flow cell primers. Libraries were sequenced on HiSeq2500 (Illumina Cambridge) for 100 cycles PE in Rapid mode. The raw sequencing data was initially processed using open source, web-based platform Galaxy (version 18.05.rc1) (https://usegalaxy.org). Reads were filtered for quality with more than 80% of the sequence having quality score > 33 using FastQC tool. Mapping against reference genome was performed with Hisat2 to the hg38 human genome. Adapter sequences were detected automatically with TrimGalore!. Reads under 20 bp were discarded. All processed sequencing files were imported in Partek® Flow® software (Partek, Inc., build 7.0.18.0514) and the gene count data was normalized using FPKM.

In the second experiment, human CD5^−^ cDC2, CD5^+^ cDC2, pDC, and pre/AS-DC from peripheral blood of *n* = 3 healthy individuals and cDC2, pDC and pre/AS-DC generated both in vivo and in vitro from *n* = 2/3 independent cord blood donors were FACS sorted. Between 100 and 1000 cells/subset were collected in TRIzol® (Thermo Fisher) and stored at -80°C. Frozen samples were shipped to GENEWIZ® where they were processed. RNA was extracted and libraries were prepared using an ultralow input RNA library preparation kit (Illumina). Libraries were sequenced on HiSeq2500 (Illumina).

The raw sequencing data was initially aligned on the human reference genome hg38 using STAR aligner (v2.5.3a)^73^ (parameters: --outFilterType BySJout --

outFilterMultimapNmax 20 --alignSJoverhangMin 8 --alignSJDBoverhangMin 1 --

outFilterMismatchNmax 999 --outFilterMismatchNoerLmax 0.04 --alignIntronMin 20 --

alignIntronMax 1000000 --alignMatesGapMax 1000000 --outSAMprimaryFlag

OneBestScore --outMultimapperOrder Random --outSAMattributes All). Raw read counts matrix was also made with STAR (using the parameter --quantMode GeneCounts).

### RNA-sequencing analysis

The average gene expression of *n* = 3 blood donors for cDC1s, *n* = 2 blood donors for cDC2 and *n* = 3 cord blood units for in vitro generated subsets were used for RNA-seq data analysis.

Hierarchical clustering was performed in Morpheus (Broad Institute, https://software.broadinstitute.org/morpheus/) using one minus Pearson’s correlation and average linkage.

GSEA (www.broad.mit.edu/gsea)^58^ was used to assess the expression of gene signatures specific for blood cDC1, cDC2, DC3, pDC, and AS-DC in in vitro and in vivo generated subsets. To simultaneously visualize pairwise comparisons of transcriptomes from cord blood-derived cDCs, the BubbleMap module of BubbleGum^59^ was used. Results were considered significant when the *p*-value was below 0.05 and the FDR (*q*) value was below 0.25. The GSEA was performed using previously published gene signatures defining blood cDC1, cDC2, DC3, pDC, and AS-DC^20^, as well as newly generated signatures using the GeneSign module of BubbleMap^59^ (Supplementary Table 6). Briefly, the transciptome of blood cDC1s and blood CD1c^+^ cells was taken from previously published datasets^60^. cDC1>CD1c^+^ and CD1c^+^>cDC1 signatures were defined using the “Min(test) vs. Max(ref)” statistical method with a minimal linear fold change = 2 and a maximal FDR = 0.01.

Heatmaps displaying the expression pattern of gene signatures for cDC1, CD1c + cells, pDC, AS-DC, and cDC2 were generated using Morpheus (Broad Institute, https://software.broadinstitute.org/morpheus/).

The 2000 genes defining the “in vitro culture imprinting” were identified using Morpheus as the mean difference of expression values between two groups: the in vitro generated cells (including cDC1, cDC2 CD206^−^ and cDC2 CD206^+^) versus ex vivo-isolated subsets (blood cDC1 and blood cDC2).

The 2872 genes defining the “in vivo signature” were identified by DEG analysis using the R package DESeq2 (version 1.24.0)^74^ with a Benjamin–Hochberg *p*-value correction^75^ (Log2FC > 1.5, adjusted *p*-value < 0.01). The volcano plot displaying the differentially expressed genes between in vivo and in vitro differentiated cDC2 was generated using R package ggplot2 (version 3.2.1)^76^ (Log2FC > 1.5, adjusted *p*-value < 0.05). All the analysis from the raw counts matrix were performed in Rstudio (1.2.5001) using the version 3.6.1 of R. Pathway analysis was performed using ConsensusPathDB (cpdb.molgen.mpg.de)^77^ and WikiPathways database.

### Mass cytometry acquisition and data analysis

For mass cytometry, pre-conjugated or purified antibodies were obtained from Invitrogen, Fluidigm (pre-conjugated antibodies), Biolegend, eBioscience, Becton Dickinson, or R&D Systems as listed in Supplementary Table 5. For some markers, fluorophore- or biotin- conjugated antibodies were used as primary antibodies, followed by secondary labeling with anti-fluorophore metal-conjugated antibodies (such as the anti-FITC clone FIT-22) or metal-conjugated streptavidin, produced as previously described^78^. Briefly, 3 × 10^6^ cells/well in a U-bottom 96-well plate (BD Falcon) were washed once with 200 µL FACS buffer (4% FBS, 2 mM EDTA, 0.05% Azide in 1× PBS) and then stained with 100 µL 200 µM cisplatin (Sigma-Aldrich) for 5 min on ice to exclude dead cells. Cells were then incubated with anti-CADM1-biotin antibody in a 50 µL reaction for 30 min on ice. Cells were washed twice with FACS buffer and incubated with 50 µL heavy-metal isotope-conjugated secondary mAb cocktail for 30 min on ice. Cells were then washed twice with FACS buffer and once with PBS before fixation with 200 µL 2% paraformaldehyde (PFA; Electron Microscopy Sciences) in PBS overnight or longer. Following fixation, the cells were pelleted and resuspended in 200 μL 1X permeabilization buffer (Biolegend) for 5 minutes at room temperature to enable intracellular labeling. Cells were then incubated with metal-conjugated anti-CD68 in a 50 µL reaction for 30 min on ice. Finally, the cells were washed once with permeabilization buffer and then with PBS before barcoding.

Bromoacetamidobenzyl-EDTA (BABE)-linked metal barcodes were prepared by dissolving BABE (Dojindo) in 100 mM HEPES buffer (Gibco) to a final concentration of 2 mM. Isotopically-purified PdCl_2_ (Trace Sciences Inc.) was then added to the 2 mM BABE solution to a final concentration of 0.5 mM. Similarly, DOTA-maleimide (DM)-linked metal barcodes were prepared by dissolving DM (Macrocyclics) in L buffer (MAXPAR) to a final concentration of 1 mM. RhCl_3_ (Sigma) and isotopically-purified LnCl_3_ was then added to the DM solution at 0.5 mM final concentration. Six metal barcodes were used: BABE-Pd-102, BABE-Pd-104, BABE-Pd-106, BABE-Pd-108, BABE-Pd-110, and DM-Ln-113.

All BABE and DM-metal solution mixtures were immediately snap-frozen in liquid nitrogen and stored at −80 °C. A unique dual combination of barcodes was chosen to stain each sample. Barcode Pd-102 was used at 1:4000 dilution, P d-104 at 1:2000, Pd-106 and Pd-108 at 1:1000, Pd-110 and Ln-113 at 1:500. Cells were incubated with 100 µL barcode in PBS for 30 min on ice, washed in permeabilization buffer and then incubated in FACS buffer for 10 min on ice. Cells were then pelleted and resuspended in 100 µL nucleic acid Ir-Intercalator (MAXPAR) in 2% PFA/PBS (1:2000), at room temperature. After 20 min, cells were washed twice with FACS buffer and twice with water before a final resuspension in water. In each set, the cells were pooled from all samples, counted, and diluted to 0.5×10^6^ cells/mL. EQ Four Element Calibration Beads (DVS Science, Fluidigm) were added at a 1% concentration prior to acquisition. Cell data were acquired and analyzed using a CyTOF Mass cytometer (Fluidigm).

The CyTOF data were exported in a conventional flow cytometry file (.fcs) format and normalized using previously described software^79^. Events with zero values were randomly assigned a value between 0 and –1 using a custom R script employed in a previous version of mass cytometry software^80^. Cells for each barcode were deconvolved using the Boolean gating algorithm within FlowJo. The CD45^+^Lin (CD7/CD14/CD15/CD16/CD19/CD34)^-^HLA-DR^+^ population of PBMC were gated using FlowJo and exported as an.fcs file. Marker expression values were transformed using the logicle transformation function^81^. Random sub-sampling without replacement was performed to select 20,000 cell events.

The transformed values of sub-sampled cell events were then subjected to UMAP dimension reduction^61,82^ using all markers. We used the 2.4.0 release of UMAP, implemented in Python, but executed through the reticulate R package to interface R objects with Python. For both mass-cytometry datasets we used UMAP using 15 nearest neighbors (*nn*), a *min_dist* of 0.2 and euclidean distance.

Heatmaps displaying mean intensity values of CyTOF data were generated using Morpheus (Broad Institute, https://software.broadinstitute.org/morpheus/).

### Human dendritic cell differentiation in vivo

Human cord blood-derived CD34^+^ hematopoietic cells (5–15 × 10^4^ cells/plug) were injected subcutaneously along with engineered stromal cells (1 : 1 to 1 : 5 ratio HSPC/MS5) in 200 μl of ice-cold Matrigel® (BD Biosciences). Mice were killed at day 12 of differentiation by cervical dislocation and Matrigel® plugs were collected. Subcutaneous Matrigel® plugs were recovered, cut in pieces and incubated in HBSS (Life Technologies) 1% FBS, 0.37 U/ml Collagenase D (Roche), 10 μg/ml DNaseI (Roche), and 1 mg/ml Dispase (Sigma-Aldrich) for 30 minutes at 37 °C. After digestion, plugs were smashed on a 100 μm strainer (Corning) and cells were collected and resuspended in FACS buffer for flow cytometry analysis.

### Histology

Matrigel plugs were fixed with 1% PFA (Alfa Aesar) for 1 hr at 4 °C, washed and incubated in 34% sucrose solution (Sigma-Aldrich) overnight at 4 °C. Plugs were embedded in Cryomatrix (Thermo Fisher) and frozen for cryostat sectioning (9 μm-thick). Sections were permeabilized using 0.5% saponin (Sigma-Aldrich), 2% BSA (Sigma-Aldrich), 1% FBS (Life Technologies) for 30 min at room temperature. For human DCs staining, plug sections were incubated with 1% rat anti-mouse CD16/32 (homemade) for 30 min to block unspecific binding sites. Sections were labeled overnight at 4 °C with mouse anti-human CD1c-PE (L161, Biolegend) or mouse anti-human Clec9A-PE (8F9, Biolegend) followed by incubation for 1 h at room temperature with goat anti-mouse Cy3 (Jackson laboratory). After extensive washes, sections were labeled with mouse anti-human CD45-APC (HI30, Biolegend) for 1 hr at room temperature. For human CD34^+^ progenitors staining, plugs sections were labeled overnight with purified mouse anti-human CD45 (HI30, Biolegend) followed by 1 hr incubation at room temperature with goat anti-mouse Cy3. After extensive washes, sections were labeled with mouse anti-human CD34^−^APC (561, Biolegend) for 1 h at room temperature. To detect murine endothelial cells, sections were labeled with purified rat anti-mouse CD31 (MEC13.3, Biolegend) and mouse anti-human CD45 (HI30, Biolegend) overnight followed by 1 h incubation at room temperature with goat anti-mouse Cy3 (Jackson laboratory) and goat anti-rat Cy5 (Jackson laboratory). All sections were labeled with Hoechst (Molecular Probes, Thermo Fisher) for nuclei staining 5 minutes at room temperature and mounted with Prolong diamond (Thermo Scientific). Slides were imaged using a SP5 (Leica Application Suite) and analyzed with Fiji software.

### Mixed lymphocyte reaction

Cord blood-derived DC subsets differentiated in vitro and in vivo were FACS-sorted into a V bottom 96-well plate (Corning) (10^4^ cells/well) and activated overnight (16 h) using a TLR agonists cocktail containing LPS 10 ng/ml, R848 1 μg/ml, and Poly(I:C) 25 μg/ml.

PBMCs were isolated by gradient centrifugation using Ficoll-Paque (GE Healthcare) and labeled with Cell Trace Violet (Thermo Fisher) according to manufacturer’s user guide. CTV-labeled T cells were then isolated using a Pan naive T-cell isolation kit (Miltenyi Biotec) according to manufacturer’s instructions and isolation purity (≥95%) was assessed by flow cytomery. Isolated naive T cells (10^5^ cells/well) were seeded together with FACS-sorted DC (1 : 10 ratio DC/T cells) and incubated at 37 °C 5% CO_2_ for 7 days.

### Statistical analysis

In all graphs each dot represents an independent cord blood donor and lines represent the median value. The number of biological as well as experimental replicates is indicated in figure legend. For each experiment, the appropriate statistical test is stated in figure legend. Statistical significance was defined as *P* < 0.05. All graphs and statistical analysis was performed using GraphPad Prism.

### Reporting summary

Further information on research design is available in the Nature Research Reporting Summary linked to this article.

## Supplementary information

Supplementary Information

Reporting Summary

## Data Availability

Data that support the findings of this study have been deposited in NCBI’s Gene Expression Omnibus (GEO) and are accessible through GEO accession codes GSE144435 [https://www.ncbi.nlm.nih.gov/geo/query/acc.cgi?acc=GSE144435] and GSE145803 [https://www.ncbi.nlm.nih.gov/geo/query/acc.cgi?acc=GSE145803].
