# Supplementary material for: Engineered niches support the development of human dendritic cells in humanized mice
Source: Nat Commun. 2020 Apr 28;11:2054. doi: 10.1038/s41467-020-15937-y (PMC7189247; doi:10.1038/s41467-020-15937-y)
Supplement: Supplementary file 1 — Supplementary Information [file 41467_2020_15937_MOESM1_ESM.pdf]

Engineered niches support the development of human dendritic cells in humanized mice

Anselmi et al.

Supplementary Information

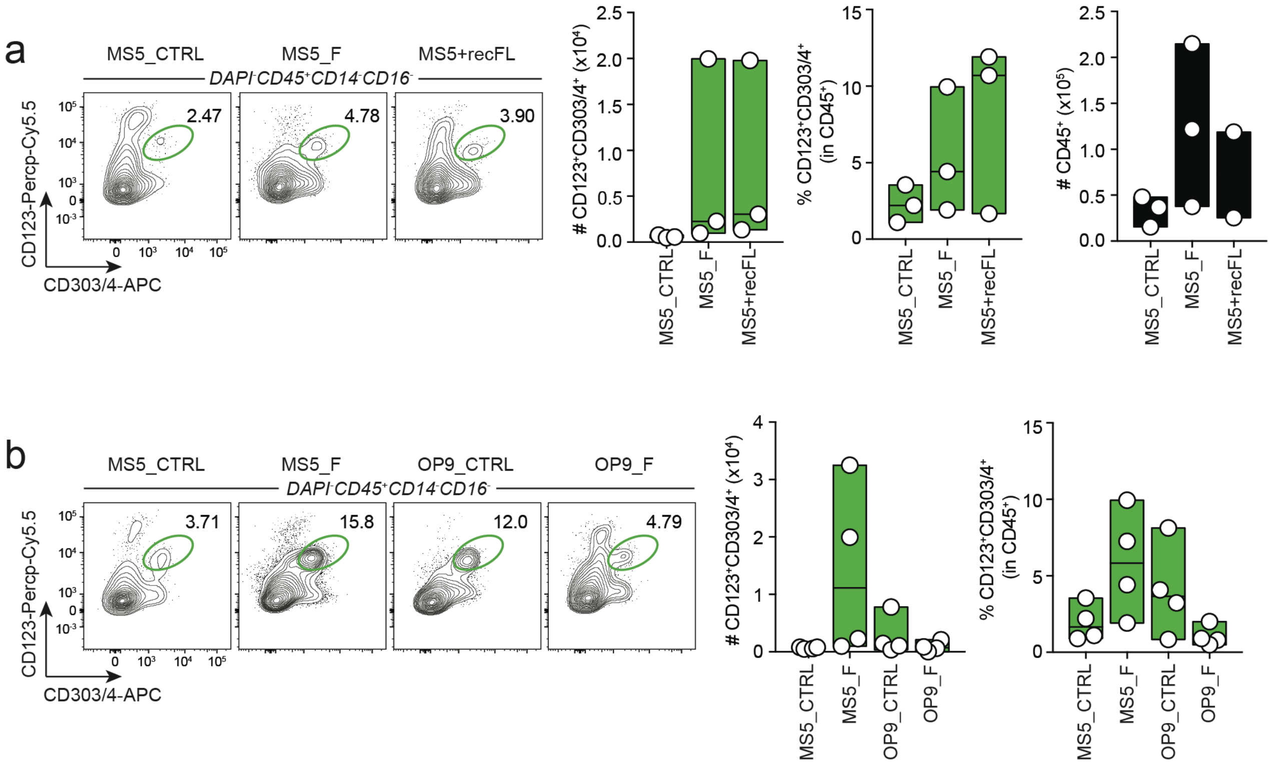

Supplementary Figure 1. Transmembrane FLT3L drives human DC differentiation *in vitro*. a Human CD123$^+$CD303/4$^+$ differentiated *in vitro* from CD34$^+$ cord blood-derived HSPCs cultured with MS5 expressing membrane bound FLT3L (MS5_F) or MS5 supplemented with recombinant human FLT3L (MS5+recFL) at day 15. n=3 donors in one experiment. One-way ANOVA test. **b** Flow cytometry plots and quantification of human CD123$^+$CD303/4$^+$ cells differentiated *in vitro* from cord blood-derived CD34$^+$ progenitors in co-culture with mouse stromal cell lines MS5 and OP9 expressing human FLT3L (MS5_F and OP9_F) at day 15. n=4 donors in one experiment. One-way ANOVA test. Data are presented as floating bars ranging from min to max and line represents median **a** and **b**.

a

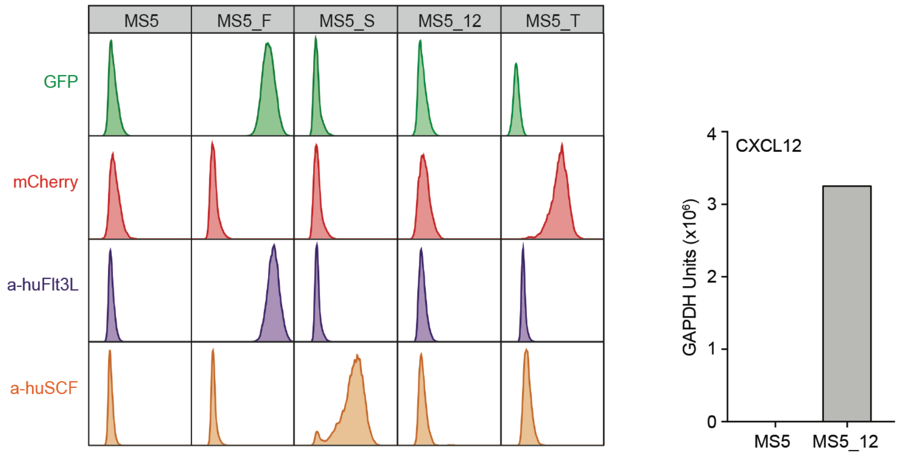

	CTRL	F	S	T	12	FS	FT	F12	ST	S12	T12	FST	FT12	FS12	ST12	FST12
huFlt3L-IRES-GFP	-	+	-	-	-	+	+	+	-	-	-	+	+	+	-	+
huSCF (no tag)	-	-	+	-	-	+	-	-	+	+	-	+	-	+	+	+
huTPO-IRES-mCherry	-	-	-	+	-	-	+	-	+	-	+	+	+	-	+	+
huCXCL12-IRES-PuroR	-	-	-	-	+	-	-	+	-	+	+	-	+	+	+	+

b

Gating strategy engineered MS5 screening:

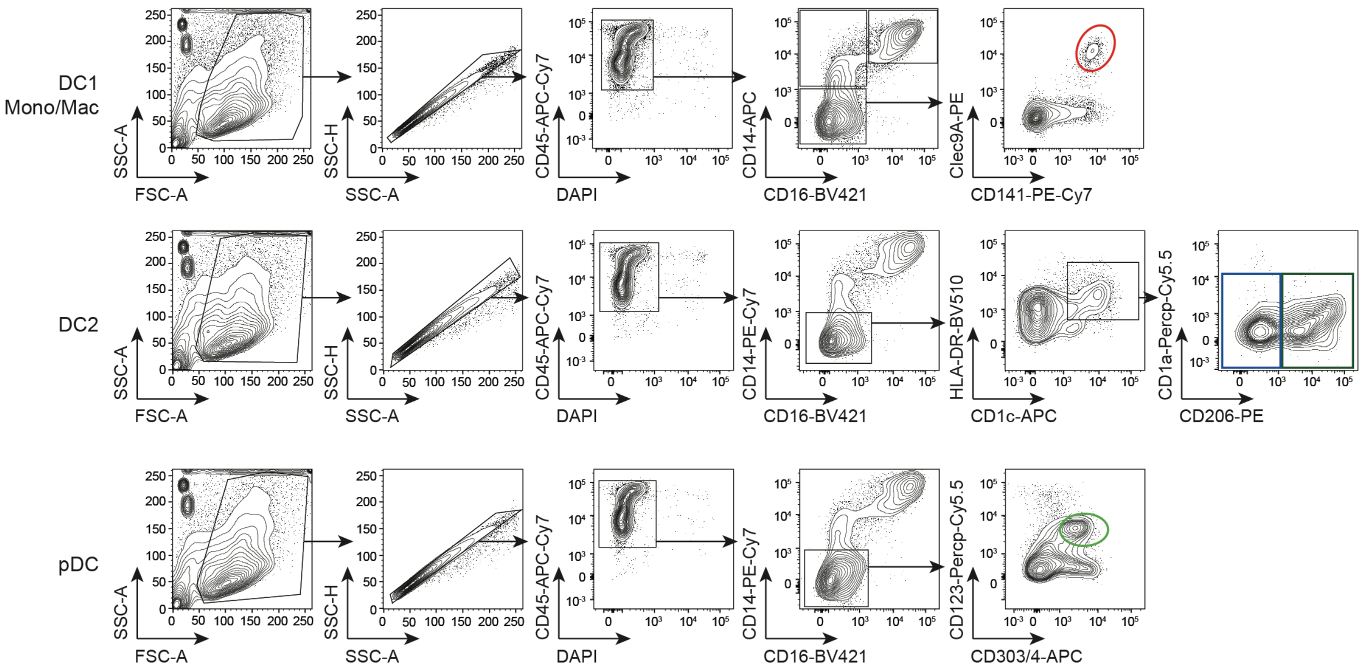

Supplementary Figure 2. CXCL12 and SCF improve FLT3L-driven DC differentiation *in vitro*. a Validation of FACS-sorted stromal lines expressing single human cytokines by flow cytometry, based on the expression of fluorescent reporters as well as antibody staining of membrane-bound FLT3L and SCF. Expression of human CXCL12 was assessed by qPCR in MS5_12 after Puromycin selection. **b** Gating strategy used to identify cord blood-derived DC subsets differentiated *in vitro* (related to figure 1, figure 2 and figure 3).

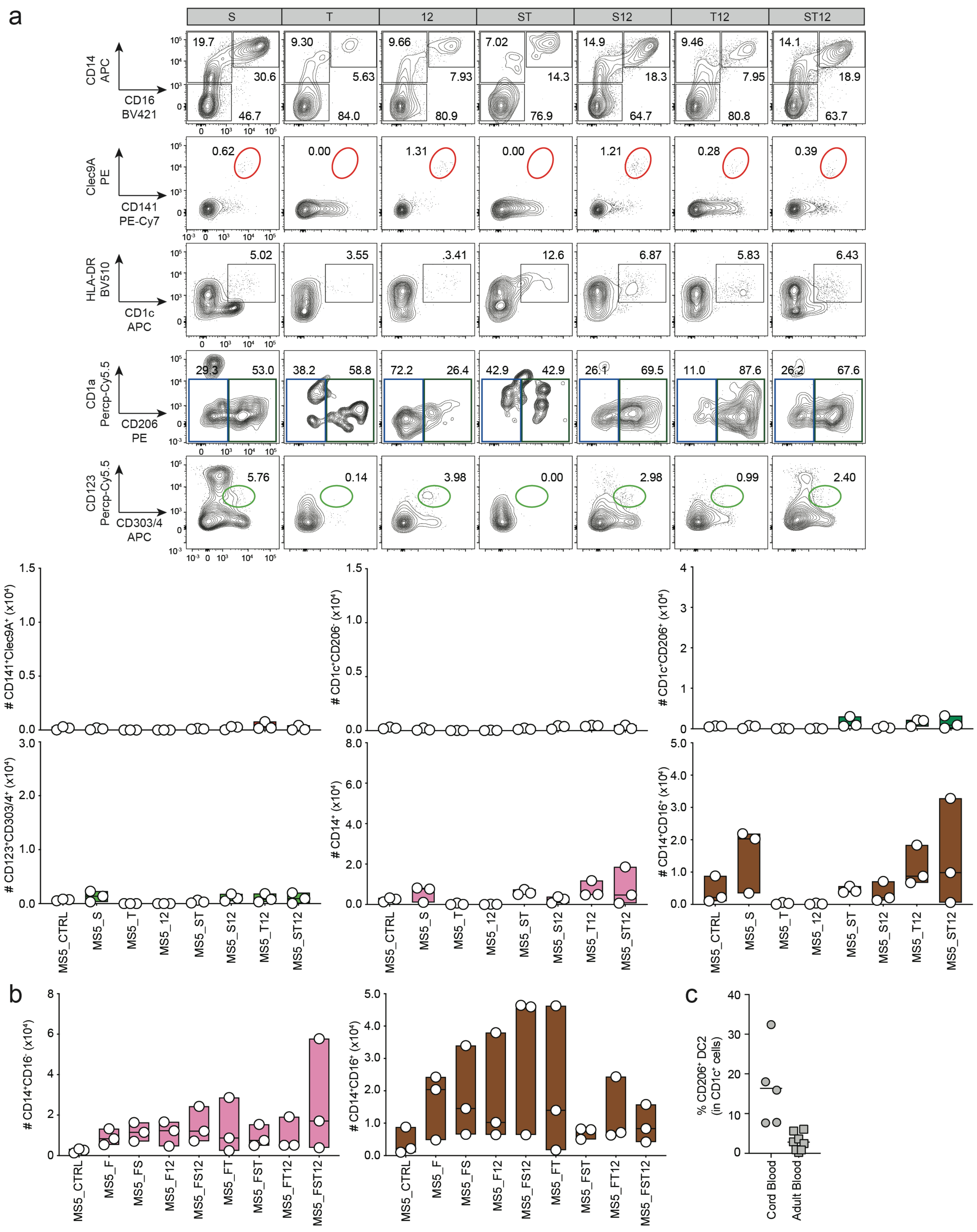

Supplementary Figure 3. CXCL12 and SCF improve FLT3L-driven DC differentiation *in vitro*. a Representative FACS plots and absolute numbers of cells generated *in vitro* from CD34^{+} HSPCs in absence of human FLT3L at day 15. n=3 cord blood donors in 3 independent experiments. **b** Absolute number of human CD14^{+}CD16^{-} monocytes and CD14^{+}CD16^{+} macrophages generated from CD34^{+} HSPCs co-cultured with MS5 expressing human FLT3L (MS5_F) in combination with human SCF (S), TPO (T) and CXCL12 (12). Day15 flow cytometry analysis of n=3 cord blood donors in 3 independent experiments. * $p<0.05$, one-way ANOVA test. **c** Frequency of CD206^{+} cells within the CD14^{-}CD1c^{+} cells in adult peripheral blood (n=8 independent donors) and cord blood (n=4 independent donors) samples. Data are presented as floating bars ranging from min to max and line represents median **a-b** or as scatter plots where line represents mean **c**.

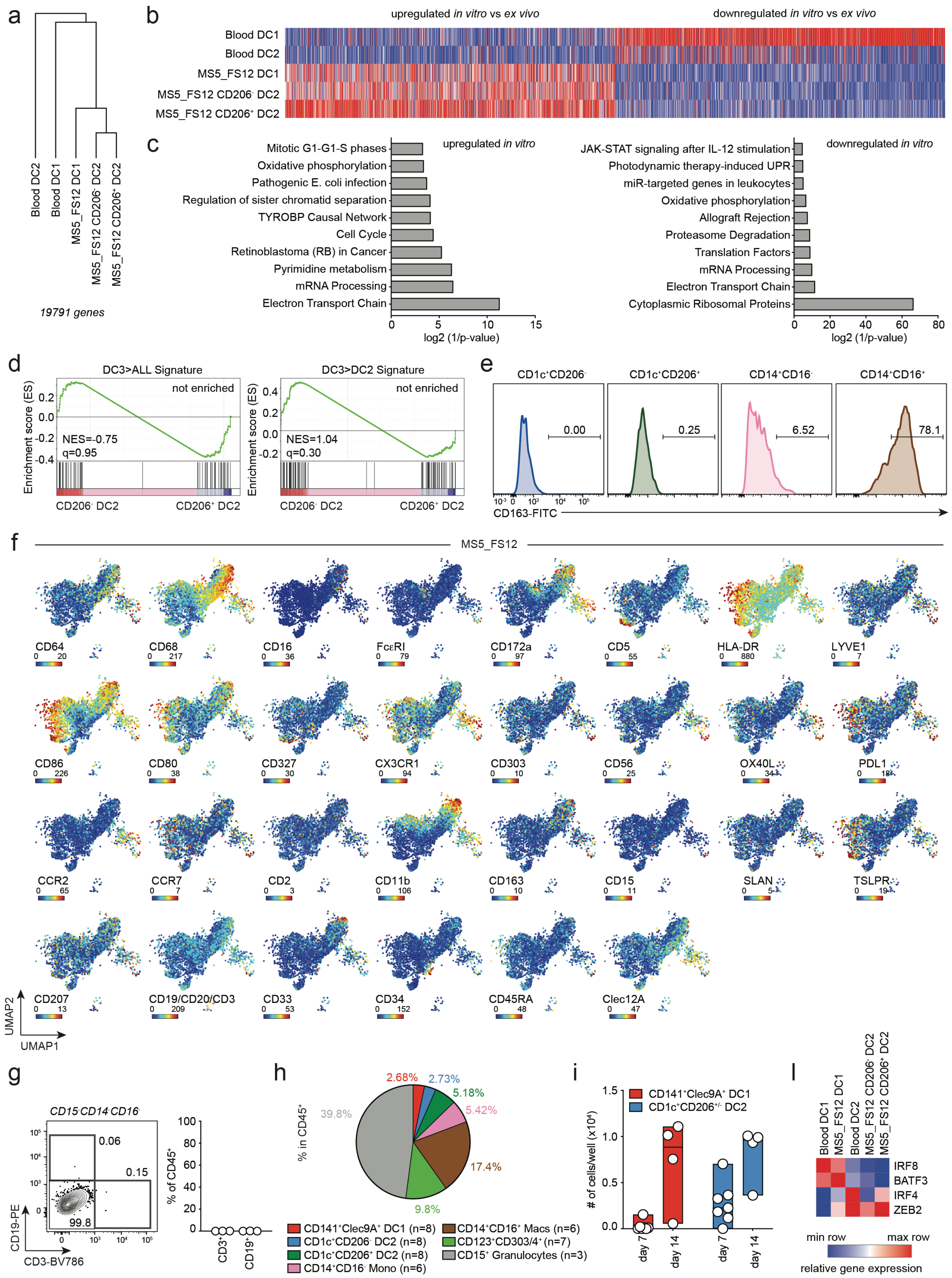

Supplementary Figure 4. Human DC generated *in vitro* align with circulating blood DC. a Hierarchical clustering of primary (n=3 healthy donors) versus *in vitro* generated (n=3 cord blood donors) cDCs based on 19791 protein-coding genes. **b** Heatmap of 2000 genes either upregulated or downregulated in *in vitro* generated cells as compared *ex vivo*-isolated blood subsets. **c** Top 10 enriched metabolic pathways (WikiPathway) for genes upregulated or downregulated in cord blood-derived cDCs generated *in vitro* as compared to *ex vivo*-isolated subsets. **d** GSEA of previously published

gene signatures (GeneSet) of blood DC3 obtained from Villani et al., Science 2017 (NES=normalized enrichment score; FDR=false detection rate). Statistical significance is defined by the FDR q value calculated by the GSEA software (www.broad.mit.edu/gsea) using default parameters. **e** Surface expression of CD163 assessed by flow cytometry in *in vitro* differentiated $CD1c^{+}CD206^{-}$ and $CD1c^{+}CD206^{+}$ cells, as well as $CD14^{+}CD16^{-}$ monocytes and $CD14^{+}CD16^{+}$ macrophages in MS5_FS12 cultures. **f** UMAP plots showing relative expression of markers detected by CyTOF in $CD45^{+}$HLA-DR^{+} cells differentiated *in vitro* using MS5_FS12. **g** Representative FACS plot and quantification of $CD3^{+}$ T cells and $CD19^{+}$ B cells generated *in vitro* using MS5_FS12. n=3 independent cord blood donors. **h** Summary of the frequency of myeloid subsets in $CD45^{+}$ cells generated *in vitro* using MS5_FS12. The number of biological replicates (n) from 3 independent experiments is shown in the figure. **i** Absolute number of cDC1 and cDC2 generated *in vitro* using MS5_FS12 at day7 (n=7) and day14 (n=4) in 3 independent experiments. **l** Relative expression of TF in blood versus *in vitro*-differentiated cDC1 and cDC2. Data are presented as floating bars ranging from min to max and line represents median **g** and **i**.

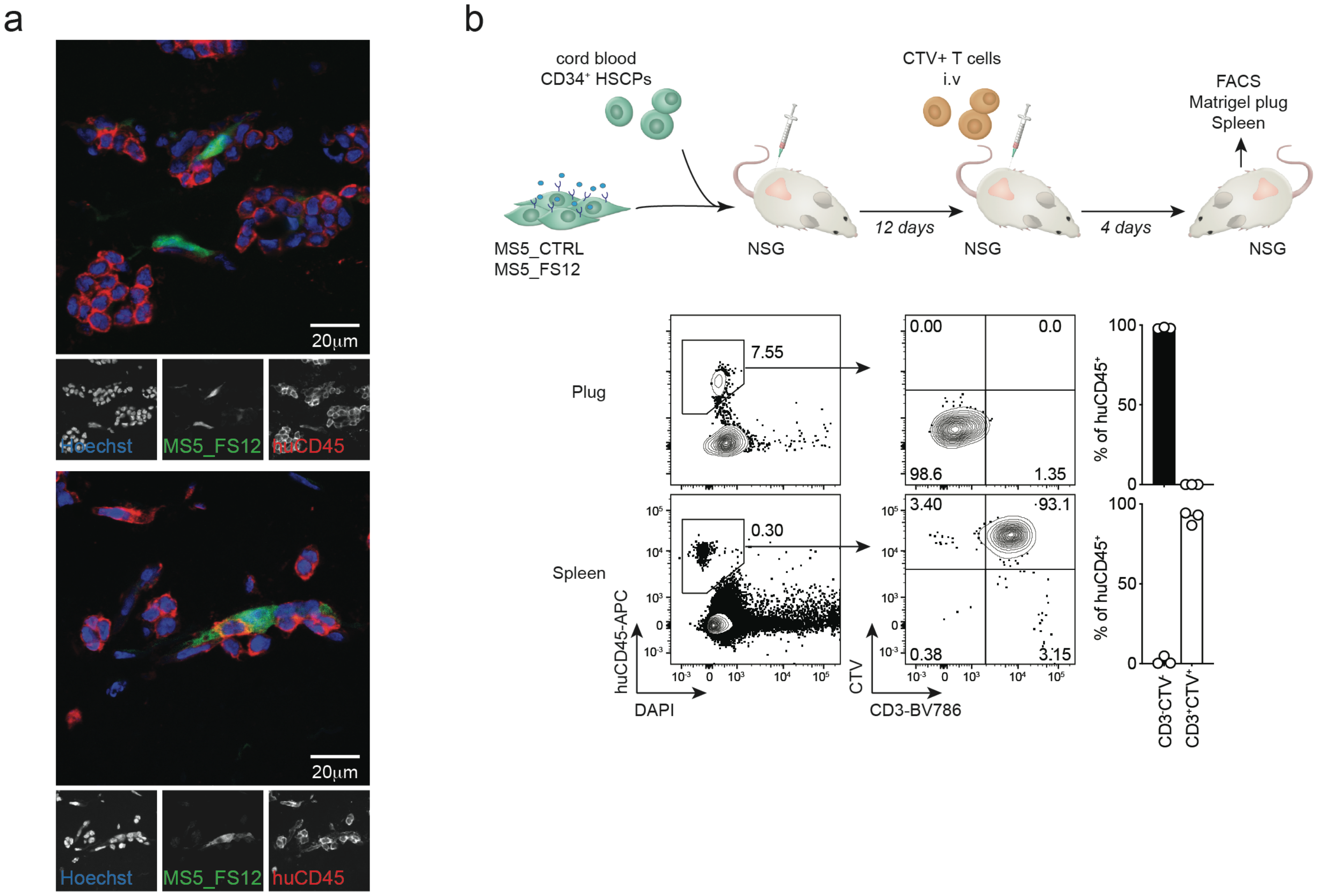

Supplementary Figure 5. *In vivo* stromal niches support HSPC maintenance and expansion. a Immunofluorescence staining of plug sections displaying the interaction of GFP+ MS5_FS12 (green) with human CD45+ cells (red). Nuclei were stained with Hoechst (blue). Arrows show interaction of human CD45+ leukocytes with GFP+ MS5_FS12. Scale bar represents 20μm. **b** Experimental strategy to assess the migration of human cells from/to MS5_FS12 plugs. CTV-labeled T cells were injected i.v. at day12 into plug-bearing mice. After 4 days, spleen and sub-cutaneous plug were recovered and the presence of human cells was assessed by flow cytometry. Mean ± SEM of n=3 mice in 1 experiment.

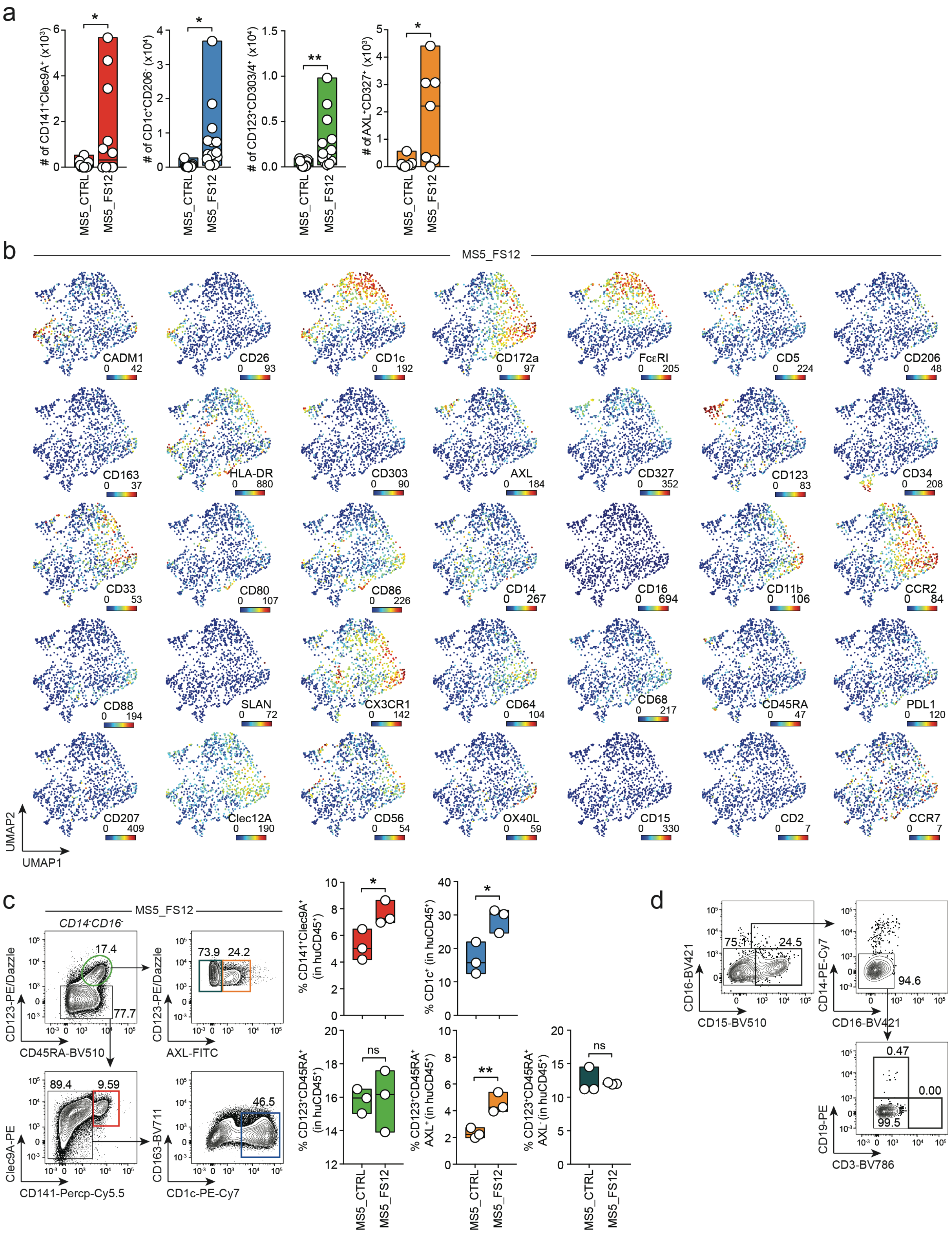

Supplementary Figure 6. The MS5_FS12 niche supports human DC development *in vivo*. a Flow cytometry analysis of Matrigel organoids containing either MS5_CTRL or MS5_FS12 stromal cells. Bar graphs show the absolute number of CD141$^+$Clec9A$^+$ cDC1, CD1c$^+$CD206$^-$ cDC2, CD123$^+$CD303/4$^+$ cells (n=14 donors in 6 experiments) and AXL+CD327+ pre/AS-DC (n=7 donors in 4 experiments). *p<0.05 **p<0.01, two-tailed paired Student t test). **b** UMAP plots showing relative expression of markers detected by CyTOF in CD45$^+$HLA-DR$^+$ cells differentiated *in vivo* using MS5_FS12. **c** Gating strategy used to identify cDC1, cDC2, pre/AS-DC, pDC and total CD123$^+$CD45RA$^+$ cells in two

physically separated plugs containing either MS5_CTRL or MS5_FS12 injected in the same recipient. Graphs summarize the frequency of each subset in total $CD45^+$ cells (n=3 cord blood donors in one experiment). * $p<0.05$, ** $p<0.01$, two-tailed paired Student t test. **d** Gating strategy used to identify human $CD15^+$ granulocytes, $CD3^+$ T cells and $CD19^+$ B cells *in vivo* in MS5_FS12 organoids. Data are presented as floating bars ranging from min to max and line represents median **a** and **c**.

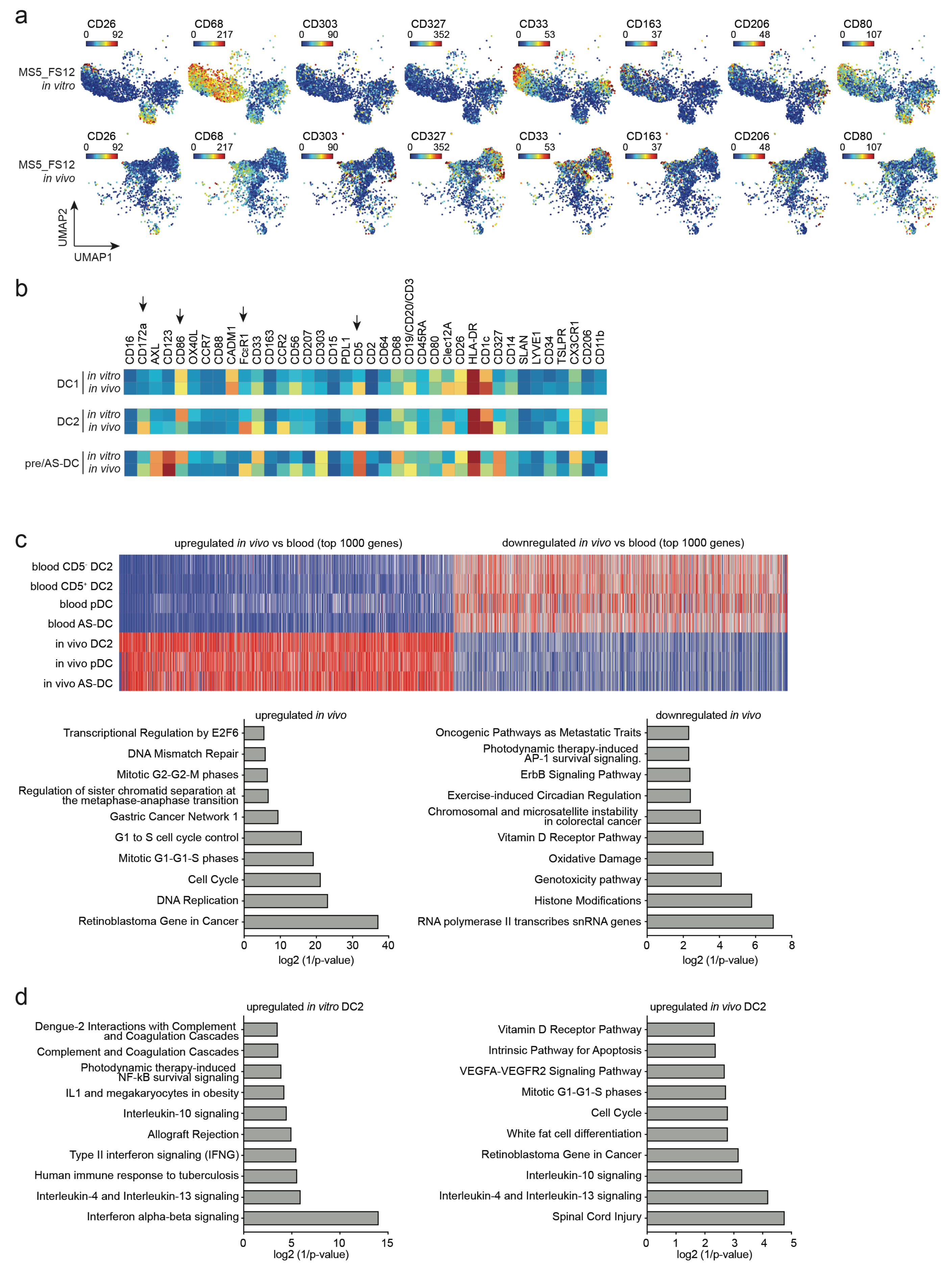

Supplementary Figure 7. cDC2 generated *in vivo* faithfully align to blood cDC2. a Relative expression of selected markers in UMAP plots of CyTOF data comparing CD45$^+$HLA-DR$^+$ cells generated using MS5_FS12 stromal cells either *in vitro* or *in vivo.* **b** Heat map of markers mean intensity (Cytof) in cDC1, cDC2 and pre/AS-DC differentiated either *in vitro* or *in vivo* using MS5_FS12 stromal cells. **c** Heatmap of top 2000 genes either upregulated or downregulated in *in*

vivo generated cells as compared *ex vivo* isolated blood subsets (top). Top 10 enriched metabolic pathways (WikiPathway) for genes upregulated or downregulated in cord blood-derived cDCs generated *in vivo* as compared to *ex vivo* isolated subsets (bottom). **d** Top 10 enriched metabolic pathways (WikiPathway) for genes upregulated in cord blood-derived cDC2 generated *in vitro* (left). Top 10 enriched metabolic pathways (WikiPathway) for genes upregulated in cord blood-derived cDC2 generated *in vivo* (right).

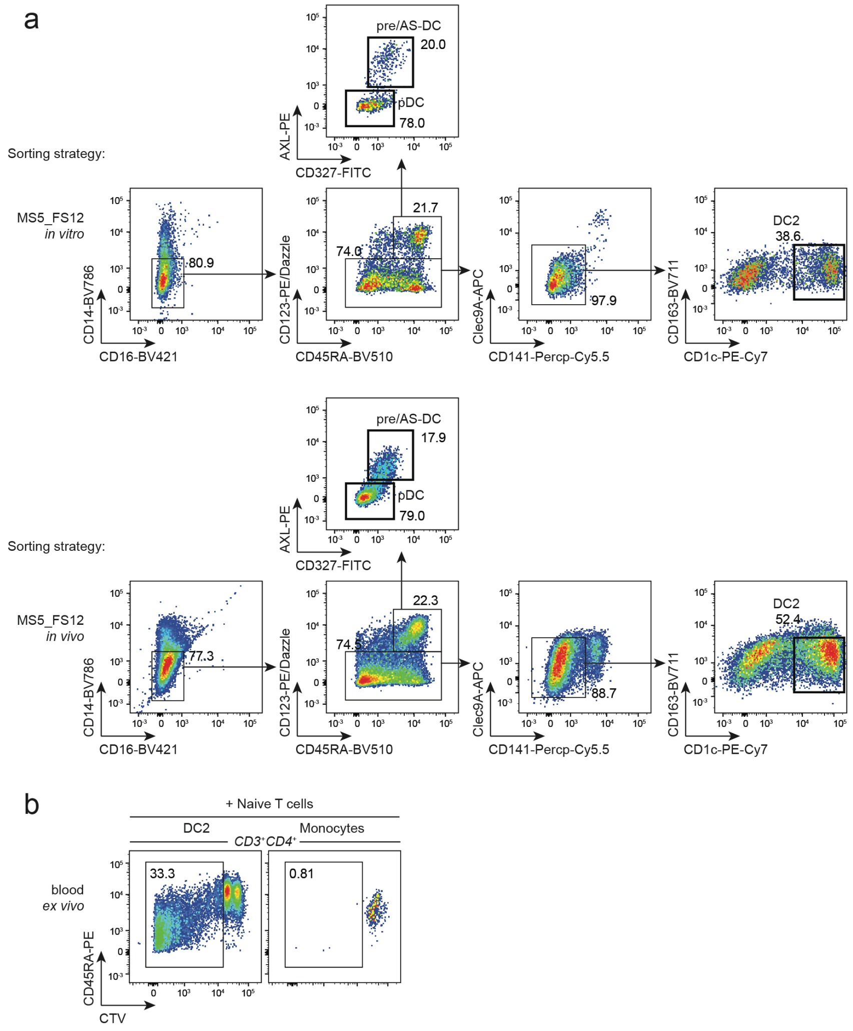

Supplementary Figure 8. *In vitro* and *in vivo* cDC2 functionally align to blood cDC2. a Gating strategy used to FACS-sort DC2, pDC and pre/AS-DC differentiated both *in vitro* and *in vivo* using MS5_FS12 stromal niche. **b** Representative FACS plots of mixed lymphocyte reaction (MLR) using *ex vivo* isolated primary cDC2 and CD14+ monocytes. FACS-sorted DC subsets were activated overnight (16 hours) using a TLR agonist cocktail (LPS 10ng/ml, R848 1μg/ml and Poly(I:C) 25μg/ml) and co-cultured with CTV-labeled naïve T cells for 5 days.

Supplementary Table 1: Human growth factor vectors

Vector	Reporter	Selection	Growth factor	cDNA source	Genebank ref.	cDNA supplier
pMX	na	Puromycin	tmSCF/KITL	pCMV6-KITL	NM_003994.4	Origene
pMX-IRES-GFP	GFP	Puromycin	tmFLT3L	pCMV6-XL5	NM_001204502.1	Origene
pMX-IRES-mCherry	mCherry	Puromycin	TPO	pMD-TPO	NM_000460.2	Origene
pBABE	na	Puromycin	CXCL12	na	na	Addgene #12270

Supplementary Table 2: Primers list

Name	Sequence (5' to 3')	Description
Flt3L Fwd	AAAAAGAATTCGCCACCATGACAGTGCTGGCGCC	to amplify Flt3L cDNA for pMX cloning
Flt3L Rev	TTTTTCTCGAGTCAGTGCTCCACAAGCAG	to amplify Flt3L cDNA for pMX cloning
tmSCF Fwd	ATATATGGATCCGCCACCATGAAGAAGACACAAACTTGGA	to amplify tmSCF cDNA for pMX cloning
tmSCF Rev	ATATATCTCGAGTTACACTTCTTGAAACTCTCTCTC	to amplify tmSCF cDNA for pMX cloning
TPO Fwd	ATATATGAATTCGCCACCATGGAGCTGACTGAATTGC	to amplify TPO cDNA for pMX cloning
TPO Rev	ATATATCTCGAGTTACCCTTCCTGAGACAGATTC	to amplify TPO cDNA for pMX cloning

Supplementary Table 3: Antibodies used in Flow Cytometry

Marker	Fluorochrome	Clone	Supplier	Cat. #			Dilution
huCD45	APC-Cy7/APC	HI30	Biolegend	304014	304012		1/100
muCD45	PerCP-Cy5.5	104	eBiosciences	45-0454-82			1/800
muCD31	A647	MEC13.3	Biolegend	102516			1/500
CD14	Pe-Cy7/BV786/APC	HCD14/M5E2	Biolegend	325618	301840	325608	1/50
CD16	BV421	3G8	Biolegend	302038			1/20
CD141	PE/PE-Cy7/PerCP-Cy5.5	M80	Biolegend	344104	344110	344112	1/20
CD1c	FITC/PE-Cy7/APC	L161	Biolegend	331518	331516	331524	1/50
Clec9A	PE/APC	8F9	Biolegend	353804	353806		1/20
HLA-DR	BV510	L243	Biolegend	307646			1/100
CD123	PerCP-Cy5.5/APC/PE_Dazzle	6H6	Biolegend	306016	306012	306034	1/50
CD303	APC	201A	Biolegend	354206			1/50
CD304	APC	12C2	Biolegend	354506			1/50
CD163	FITC/BV711	GHI/61	Biolegend	333618	333630		1/50
CD206	PE/APC/PerCP-Cy5.5	15.2/6H6	Biolegend	321106	321110	321122	1/50
CD3	BV786/FITC	OKT3	Biolegend	317330	317306		1/100
CD327/Siglec6	FITC	767329	R&D	FAB2859G			1/20
Axl	PE/FITC	108724	R&D	FAB164P			1/20
CD15	BV510/BV421	W6D3	Biolegend	323028	323040		1/50
CD19	PE	HIB19	Biolegend	302208			1/50
CD34	APC/FITC	561	Biolegend	343608	343604		1/50
CD45RA	BV510/PE	HI100	Biolegend	304142	304108		1/100
α-tmSCF	biotin	polyclonal	R&D	AF-255-NA			1/50
α-Flt3L	biotin	polyclonal	R&D	AF-308-NA			1/50
CD1a	APC/FITC/Percp-Cy5.5	HI149	Biolegend	300110	300104	300130	1/50
NKp46	biotin	9E2	Biolegend	331906			1/10
CD3	biotin	OKT3	Biolegend	317320			1/50
CD19	biotin	HIB19	Biolegend	302204			1/100
CD20	biotin	2H7	Biolegend	302350			1/100
CD15	biotin	MC-480	Biolegend	125604			1/50
CD203c	biotin	FR3-16A11	Miltenyi Biotec	130-092-345			1/50
Streptavidin	APC-Cy7	-	Biolegend	405208			1/400
IFNα	PE	7N4-1	BD Bioscience	560097			1/50
IL-12	PE	C11.5	BD Bioscience	559329			1/50
TNFα	AF700	SE5A5	Biolegend	502928			1/50
CD4	APC-Cy7	A161A1	Biolegend	357416			1/50
CD8	APC	HIT8a	Biolegend	300912			1/100
CD45RO	PerCP-Cy5.5	UCHL1	Biolegend	304252			1/50
CD86	PE	BU63	Biolegend	374206			1/100
CD83	FITC	HB15e	Biolegend	305306			1/20

Supplementary Table 4: Antibodies used in Immunofluorescence and Neutralizing antibodies

Immunofluorescence					
**Marker**	**Fluorochrome**	**Clone**	**Supplier**	**Cat. #**	**Dilution**
murine CD31	A647	MEC13.3	Biolegend	102516	5μg/ml
CD1c	PE	L161	Biolegend	331506	10μg/ml
Clec9A	PE	8F9	Biolegend	353804	10μg/ml
CD34	APC	561	Biolegend	343608	10μg/ml
CD45	purified	HI30	Biolegend	304002	5μg/ml
CD45	APC	HI30	Biolegend	304037	5μg/ml
anti-mouse	Cy3	polyclonal	Jackson Immunoresearch	115-165-166	2.5μg/ml
anti-mouse	Cy5	polyclonal	Jackson Immunoresearch	115-175-166	2.5μg/ml

Neutralizing Antibodies					
**Marker**	**Fluorochrome**	**Clone**	**Supplier**	**Cat. #**	**Dilution**
human GM-CSF	unconjugated	polyclonal	R&D	AF-215-SP	2μg/ml
Goat IgG	unconjugated	polyclonal	R&D	AB-108-C	2μg/ml

Supplementary Table 5: Antibodies used in Mass Cytometry

Marker	Metal	Clone	Supplier	Cat. #
CD45	Y89Di	HI30	Fluidigm	3089003B
CD14	Cd112Di	TuK4	Invitrogen	MHCD1400
CD15	In115Di	hi98	Biolegend	301902
Clec12A	Pr141Di	50C1	R&D Sysytems	MAB2946
CD5	Nd142Di	UCHT2	Biolegend	C 300602
CD2	Nd143Di	RPA-2.10	Biolegend	300202
CD64	Nd144Di	10.1	Biolegend	305002
CD68	Nd145Di	Y1/82A	eBioscience	14-0688-80
CD19	Nd146Di	HIB19	eBioscience	14-0199-82
CD20	Nd146Di	2h7	eBioscience	14-0209-82
CD3	Nd146Di	ucht1	Biolegend	317302
CD26	Sm147Di	BA5B	Biolegend	302702
CD45RA	Nd148Di	HI100	Biolegend	304102
HLA-DR	Sm149Di	L243	Biolegend	307602
CD80	Nd150Di	L307.4	BD Biosciences	557223
CADM1	Eu151Di	3E1	MBL	CM004-3
CD1c	Sm152Di	L161	Biolegend	331502
FcER1	Eu153Di	AER-37	eBioscience	14-5899-82
CD327	Sm154Di	767329	R&D systems	MAB2859
CD33	Gd155Di	wm53	BD Biosciences	555449
CD163	Gd156Di	GHI/61	Biolegend	333602
CCR2	Gd157Di	k036c2	Biolegend	357202
CD56	Gd158Di	NCAM16.2	BD Biosciences	559043
SLAN	Tb159Di	DD-1	Miltenyi Biotec	130-093-031
CD207	Gd160Di	DCGM4/122D5	Novus Biologicals	DDX0363P-100
CD172a	Dy161Di	SE5A5	Biolegend	323902
AXL	Dy162Di	MM0098-2N33	Novus Biologicals	MM0098-2N33
CD123	Dy163Di	6h6	BD Biosciences	554527
CD303	Ho165Di	201A	Biolegend	354202
CD86	Er166Di	IT2.2	BD Biosciences	555663
OX40L	Er167Di	11C3.1	Biolegend	326302
CCR7	Er168Di	mab197	R&D systems	MAB197-100
LYVE1	Tm169Di	af2089	R&D systems	AF2089
CD88	Er170Di	S5/1	Biolegend	344302
CD34	Yb171Di	581	Biolegend	343502
TSLPR	Yb172Di	1B4	Biolegend	322802
CX3CR1	Yb173Di	K0124E1	Biolegend	355702
CD206	Yb174Di	15.2	Biolegend	321102
PDL1	Lu175Di	29E.2A3	Biolegend	329719
CD11b	Yb176Di	ICRF44	Biolegend	301302
DNA	Ir191Di			
DNA	Ir193Di			
Cisplatin	Pt195Di			
CD16	Bi209Di	3G8	Biolegend	302002

Supplementary Table 6: Gene signatures used in GSEA analysis
(referring to Fig. 3c, Fig.4c, Supplementary Fig.4d and Fig. 6d)

DC1>CD1c+[1]	CD1c+>DC1[1]	DC1>ALL[2]	DC2>ALL[2]	DC2>DC3[2]	DC3>DC2[2]	DC3>ALL[2]	AS-DC[2]	pDC[2]
ADRA1A	ACOT11	CLEC9A	CD1C	HLA-DQB	AK307192	S100A9	AXL	GZMB
ANO9	ACTA2	C1orf54	FCER1A	HLA-DPB1	BACH1	S100A8	PPP1R14A	IGJ
AP3M2	ADAM11	HLA-DPA1	CLEC10A	HLA-DQB1	CA5BP1	VCAN	SIGLEC6	AK128525
APOL1	ADAP1	CADM1	ADAM8	HLA-DQA1	TSC2	LYZ	CD22	SERPINF1
APOL2	ADCYAP1	CAMK2D	CD1D	HLA-DQA2	SHOC2	ANXA1	DAB2	ITM2C
APOL3	JAML	CPVL	FCGR2B	DQ-A1	HPCAL1	PLBD1	S100A10	PLD4
ARHGAP12	ARSA	WDFY4	CLEC4A	CD1C	PVR	RNASE2	FAM105A	CCDC50
BANF1	ATP6V0A1	CPNE3	SLC2A3	HLA-DOB	RIPK2	FCER1A	MED12L	IRF7
BCL6	BTBD11	IDO1	CD33	P2RY14	STIM1	SLC2A3	ALDH2	PTPRS
BIK	LAMP5	HLA-DPB1	ETS2	ARL4C	ID1	CD163	LTK	ALOX5AP
BTLA	CPED1	HLA-DOB	CLIC2	CLIC2	IKBKE	CSF3R	DPYSL2	TCF4
C10orf105	CACNA2D3	HLA-DQB1	PEA15	CLEC17A	KCNN4	MNDA	LGMN	BCL11A
RUBCNL	CAPN3	CLNK	CACNA2D3	C10ORF128	EMP1	CD14	IRF4	LILRA4
LACC1	CD1A	CSRP1	CD1E	FAM26F	LPPR2	NAIP	SEPT6'	PLAC8
C1orf115	CD1B	SNX3	MBOAT7	ASAP1	GPBAR1	CSTA	PLAC8	C12ORF75
C1orf186	CD1C	ZNF366	C10orf128	SLC41A2	LOC284454	FCN1	CCND3	FAM129C
C1orf21	CD1E	SHTN1	NR4A2	SLAMF7	MKNK1	CD1D	MYO1E	CYBASC3
CIART	CD2	NDRG2	GPAT3	CST7	KIAA0513	FPR1	SLC41A2	MZB1
C1orf54	CD33	ENPP1	ENTPD1	PKIB	FOXO3	F13A1	SCN9A	UGCG
ACTL10	CFP	RGS10	CD2	HSPA7	TMEM111	CLEC10A	SIGLEC1	DERL3
ROMO1	CHD3	CYB5R3	PER1	CXCL16	YWHAG	CES1	CX3CR1	IL3RA
GFOD1	CLEC10A	ID2	PID1	RUNX3	ECRP	PID1	NDRG1	SPIB
ERICH5	CLEC4A	XCR1	AREG	WDFY4	TAB1	S100A12	VASH1	ZFAT
CADM1	CLEC4F	CCSER1	PTGS1	IL18R1	OSM	MTMR11	CD5	SMPD3
CAMK2D	CCP110	ASAP1	SMN1	FCGR2B	GABARAPL1	SMA	BHLHE40	NRP1
CASP2	CREB3L2	SLAMF8	CLEC17A	MYO1E	ASPH	LAT2	SNRNP25	TSPAN13
CBL	CRYZL1	CD59	ITGA5	AXL	PDLIM7	RETN	USF2	LIME1
CCDC127	CTSW	DHRS3	CREB5	PEA15	QPCT	TMEM173	SLC20A1	CLEC4C
MCUR1	DAGLB	GCSAM	PTAFR	SIGLEC10	RIN2	AOAH	ATF5	CLIC3
CCND1	DENND1A	FNBP1	NOD2	CD1E	MRPS23	RAB3D	FAM129A	SPCS1
CCR9	DHRS9	TMEM14A	CCR6	GOLGA8B	PLXND1	CD36	KLF4	NPC1
CD226	EAF2	NET1		IFITM1	CLEC12A	MGST1	RUNX2	HIGD1A
CDK2AP1	EFNB1	BTLA		LOC100505746	TMEM176A	TREM1	ARHGAP18	CTSB
CDK2AP2	ENHO	BCL6		FEZ1	PISD	HNMT	APEX1	NPC2
CLEC4C	EPB41L2	FLT3		INSIG1	PLA2G7	CES1P1	ENTPD7	SEC61B
CLEC9A	EVI5	ADAM28		SPATS2L	TMEM141	ADAM15	SLC35C2	C1ORF186
CLNK	FCER1A	SLAMF7		GRIP1	NINJ1	IL13RA1	CDH1	TNFRSF21
CLSTN2	FCGBP	BATF3		MCOLN2	AGTRAP	MICAL2	GPR146	IRF8
COX15	FCGR2B	LGALS2		SERTAD3	BLVRA	ITGA5	BAIAP2	HERPUD1
CSRP1	FCRLB	VAC14		PPP1R14A	HBEGF	CREB5	CDKN1A	PLP2
CTNND2	FGD4	PPA1		UVRAG	DMXL2	IL1B	UPK3A	SLC15A4
CYB5R3	FXYD5	APOL3		SIGLEC6	C9ORF89	NR4A2	GNAQ	CD164
CYYR1	GAS1	C1orf21		KPNA6	IL1B	MPP7	THBD	BLNK
DBN1	GAS2L1	CCND1		LGMN	NLRP12	PTAFR	TNFSF12	NCF1C
FAM149A	GYPE	ANPEP		SPIB	SORL1	HBEGF	SOX4	HSP90B1
DPP4	ITGAX	ELOVL5		SNURF-SNRPN	NFE2	NFE2	CXCR2	OGT
DSTN	ITPR1	NCALD		LOC645638	ADAM15	ASGR1	HIP1	SELS
DYSF	JUP	ACTN1		TOP1MT	CCDC69	BST1	STX18	IRF4
EIF5B	KCTD5	PIK3CB			SULT1A1	IL1RN	CTSW	APP
ENOX1	LAYN	HAVCR2			TOM1	NOD2	ATP2B4	TXN
ENPP1	NRROS	GYPC			KCNE3	NLRP3	CD72	RUNX2
FAM102A	NAA38	TLR10			PYGL	DQ575504	MGLL	PTPRCAP
FAM160A2	C1orf228	ASB2			SLC11A1	LMNA	SUSD1	GPR114
TOGARAM2	MTMR11	KIF16B			HK3	C9ORF89	RNF141	STMN1
FAM53A	MYO1A	LRRC18			ACSL1	IL27RA	TNNI2	RNASE6
FAM53C	MYO5C	DST			IER3	NLRP12	GGTA1P	PFKFB2
FAR2	NOD2	DENND1B			CFD	RAB27A	C5ORF25	MAP1A
FARP2	OLFM1	DNASE1L3			LMNA	EREG	PTGDS	NUCB2
FASTKD5	OSGEP	SLC24A4			SEPX1	LOC284454	TSEN54	SSR4
FBXO31	OXER1	VAV3			TREM1		KLF12	LAMP5
FLNB	PIK3C2A	THBD			PILRA		MYH11	NCF1
PGAP2	PIP4K2A	NAV1			ASGR1		TXN	B4GALT1
FUCA1	PPP1R16A	GSTM4			TXNRD1		AK125727	IGFLR1
FUT8	PRKCA	TRERF1			GLUL		CD300LB	NOTCH4
GCSAM	PRKCE	B3GNT7			PSTPIP1		SUCLA2	GPR183
GNAO1	PROCR	LACC1			CSF3R		BIN1	EPHB1
GRAP2	PTAFR	LMNA			STAB1		MRPS6	LOC285972

Notes:

1- Generated using BubbleGum software from McGovern et al. dataset [1]

2- Discriminative genes for each subset reported in Villani et al. [2]

DC1>CD1c+[1]	CD1c+>DC1[1]	DC1>ALL[2]	DC2>ALL[2]	DC2>DC3[2]	DC3>DC2[2]	DC3>ALL[2]	AS-DC[2]	pDC[2]
GRAP2	PTAFR	LMNA			STAB1		MRPS6	LOC285972
HTR3A	PTGS1	PTK2			RETN		ZNF789	MYBL2
ICOSLG	RNASE2	IDO2			SERPINA1		RAD1	PTCRA
ISYNA1	RNF130	MTERF3			SLC7A7		PIM2	SLA2
KATNA1	RTN1	CD93			CTSD		PLA2G16	AK093551
FAM131B	S100A4	DPP4			NEAT1		TBC1D9	PLXNA4
ARHGAP39	SEC24B	SLC9A9			CES1P1		ADAM33	SEPT1'
LRRC1	SIAH3	FCRL6			FPR1		ZEB1	C10ORF118
CARMIL1	SIDT1	PDLIM7			CD163		CD300LG	LILRB4
MAGEE1	SIGLEC5	CYP2E1			S100A12		SLC4A3	GAPT
MPRIP	SNCA	PDE4DIP			CYBB		STAG3L4	IDH3A
MRE11	SNN	LIMA1			F13A1		MECR	MS4A6A
MRPL18	SNRK	CTTNBP2NL			CES1		COQ7	FMNL3
MSL3	TMEM173	PPM1M			BST1		RBL1	SNRPN
NAPEPLD	TMEM39B	OSBPL3			MTMR11		CEP95	KIAA0226L
BEX3	TMEM71	PLCD1			CD36		RNASEL	BC051760
NUDT11	TMEM86A	CD38			MGST1		ACPP	ST6GALNAC4
OSBP2	TNFRSF10D	EHD4			RAB3D		SP4	OFD1
PACRG	TRIB2	ACSS2			PLBD1		LAX1	C9ORF142
PAPSS1	TSEN34	FUCA1			TMEM176B			TGFBI
PDE6D	TSGA10IP	SNX22			CD14			SELL
PKP4	TSNAX	APOL1			FCN1			SIDT1
PLEK	VSIG4	DUSP10			RNASE2			TRAF4
PLEKHA2	ZBP1	FAM160A2			VCAN			DCK
PLXNA1	ZNF175	INF2			S100A8			ERN1
PLXNB1	ZNF23	DUSP2			S100A9			TPM2
PNLDC1	CD200R1	PALM2						PARK7
POLA2	PIP4K2A	RAB11FIP4						TLR7
PLPP1	ROBO1	DSE						CARD11
PPM1M	CHD1	FAM135A						DAB2
PPT1	TEP1	KCNK6						ERP29
PPY	ABL1	PPM1H						PACSIN1
PRKCZ	BRD3	PAFAH1B3						LOC644961
PTK2	CSHL1	PDLIM1						RABGAP1L
QPRT	FAT4	TGM2						ADAM19
RAB7B	CCDC136	SCARF1						SORL1
RCOR2	PPFIA4	CD40						PPP1R14B
ROMO1	ASB16	STX3						SCAMP5
RUSC1	CRH	PRELID2						USP24
SEC24D	ZMYM6	PQLC2						ZDHHC17
SEMA4F	AKAP14							CXCR3
SEPHS2	CD207							MAN2B1
SERTAD3	CLDND2							RNASET2
SH3RF2	MINDY1							FCHSD2
SHE	ASIC1							LAIR1
SLAMF7								OVOS2
SLC16A9								P2RY14
SLC24A4								CYTH4
SLC25A23								PPM1K
SLC46A3								ABHD15
SLC9A9								EIF4A3
SMO								P4HB
SNRPN								NCF1B
SNX22								TSPAN3
SNX3								TRAM1
ST7								ABPARTS
SYPL1								COBLL1
TACSTD2								CREB3L2
TANC2								TMEM109
TAP2								SCN9A
TDRD10								CYP46A1
THBD								LGMN
TIMELESS								NGLY1
TMEM106C								C17ORF109
SLC35G1								PLA2G16
TRIP6								SLC38A1
TSPAN2								PHEX
VAC14								CD99
VCX3A								PPM1J

Notes:

1- Generated using BubbleGum software from McGovern et al. dataset [1]

2- Discriminative genes for each subset reported in Villani et al. [2]

DC1>CD1c+[1]	CD1c+>DC1[1]	DC1>ALL[2]	DC2>ALL[2]	DC2>DC3[2]	DC3>DC2[2]	DC3>ALL[2]	AS-DC[2]	pDC[2]
WASF3								C10ORF58
ZNF239								KIAA0226
ZNF711								DHRS7
CCDC113								CNP
ITPRIPL1								CDCA7L
TH								SIT1
CCDC62								TACC1
DPY19L2								RASD1
EPB41L1								TMIGD2
MCFD2								KRT5
NLRP4								ASPH
RASSF2								LOC652276
TRIB3								PDIA4
VASH1								AHI1
ACP5								GPM6B
TMEM237								HPS4
CAPN12								SIVA1
PHKA1								LOC100507600
ZFP1								UBE2J1
ADAD2								FAM160A1
AIM2								IFI44L
AKTIP								MAPKAPK2
CEP70								CMKLR1
CLEC1A								AX747844
DGAT2								GGA2
KIAA1958								TP53I13
NDRG4								CSF2RB
MAEL								LOC100233209
MTRR								TCL1A
TM9SF2								ATP2A3
ZNF462								FLNB
SERAC1								NEK8
FBXO27								TBC1D4
TGM5								CUX2
								PDCD4
								SND1
								SLC2A1
								SMC6
								LY9
								STAMBPL1
								KIRREL3
								SCARB2
								EMB
								PAFAH2
								VEGFB
								AL833181
								DQ572107
								ZCCHC11
								DUSP5
								SLC38A2
								SLC7A5
								TTC24
								ANKRD36
								TMEM19
								LOC100131564
								CD2AP
								GAS6
								IGFBP3
								MIF4GD
								IRF2BP2
								CRYM
								DKFZP586I1420
								DKFZP667P0924
								TEX2
								FLJ43663
								FKBP2
								SPICE1
								AHNAK2
								ANKRD36BP1

Notes:

1- Generated using BubbleGum software from McGovern et al. dataset [1]

2- Discriminative genes for each subset reported in Villani et al. [2]

DC1>CD1c+[1]	CD1c+>DC1[1]	DC1>ALL[2]	DC2>ALL[2]	DC2>DC3[2]	DC3>DC2[2]	DC3>ALL[2]	AS-DC[2]	pDC[2]
								RNF5
								RRBP1
								SLC12A3
								SLC3A2
								SEC61G
								ATP13A2
								LRRC36
								AK095700
								C12ORF44
								POLB
								LMAN1
								AK057596
								PHC3
								SUSD1
								ANKRD36B
								CRIM1
								MGAT4A
								SEL1L3
								SLC7A11
								MILR1
								PAPLN
								CLN8
								VAMP1
								CCDC69
								KANK1
								LTB
								STRBP
								SLC20A1
								SNURF-SNRPN
								SOLH
								PARP10
								BX647938
								PAIP1
								MAGED1
								DHTKD1
								IL28RA
								C5ORF62
								SLC35E2
								FZD3
								EGLN3
								MEF2D
								TNFAIP3
								COL24A1
								MCOLN2
								TUBB6
								CLCN5
								FUT7
								SFT2D2
								CSNK1E
								NOP56
								ST3GAL4
								DPPA4
								GNG7
								SEC61A1
								DSN1
								FLJ42627
								ZDHHC4
								CCR2
								C6ORF25
								ITPR2
								TMEM63A
								ABCA2
								ADA
								FOXRED2
								ST3GAL2
								PMS2P5
								SGSM3
								USP11
								GAB1

Notes:

1- Generated using BubbleGum software from McGovern et al. dataset [1]

2- Discriminative genes for each subset reported in Villani et al. [2]

DC1>CD1c+[1]	CD1c+>DC1[1]	DC1>ALL[2]	DC2>ALL[2]	DC2>DC3[2]	DC3>DC2[2]	DC3>ALL[2]	AS-DC[2]	pDC[2]
								STT3A
								SULF2
								C18ORF8
								DENND5B
								NFX1
								SUZ12P
								CTNS
								TXNDC5
								SETBP1
								TATDN3
								LOC642776
								MDFIC
								SEC11C
								UBA5
								MYO1E
								TASP1
								PIK3CD
								MDN1
								PPARA
								DQ576756
								TCL6
								TGFBR2
								TP53I11
								11-Sep
								SBDS
								ZFYVE26
								BTAF1
								C5ORF45
								PTK7
								SRPR
								ERO1LB
								NAPSA
								C9ORF91
								STAG3L3
								TULP4
								CYSLTR1
								LOC284551
								SNRNP25
								ALG2
								ITGAE
								MAP2K6
								TBCC
								OCLN
								DCPS
								LRP8
								STAG3L1
								KRR1
								C12ORF45
								PCYOX1
								SPNS3
								TPST2
								MYB
								SLC12A2
								ZBTB33
								ABI2
								PMS2L2
								GLCE
								ITPR1
								MRPL36
								C5ORF64
								PFKP
								S100PBP
								SPON2
								SPG20
								TRDMT1
								N4BP2L1
								PPP6R1
								RCL1
								ZNF506

Notes:

1- Generated using BubbleGum software from McGovern et al. dataset [1]

2- Discriminative genes for each subset reported in Villani et al. [2]

DC1>CD1c+[1]	CD1c+>DC1[1]	DC1>ALL[2]	DC2>ALL[2]	DC2>DC3[2]	DC3>DC2[2]	DC3>ALL[2]	AS-DC[2]	pDC[2]
								AHCY
								CXORF21
								CCS
								RNASEH2B
								SYS1
								P2RY6
								PPFIBP1
								NFATC2IP
								ZNF527
								MINA
								TAX1BP3
								DAAM1
								GALNT2
								LOC400657
								C1ORF55
								RREB1
								VIPR2
								ARL6IP6
								QDPR
								ABCA7
								SLC23A2
								BEX4
								SLC33A1
								THSD1P1
								ARHGEF4
								C6ORF170
								N4BP2
								SPATA5
								CRYM-AS1
								IQGAP2
								DAPK2
								MFSD2A
								PCMTD1
								ANKS3
								CEP135
								LOC100131089
								ALDH5A1
								BC034268
								MAP4K4
								SERTAD2
								PCNX
								PHLPP2
								EFHC1
								SP4
								TRRAP
								NICN1
								TRIM74
								HNRNPA1L2

Notes:

1- Generated using BubbleGum software from McGovern et al. dataset [1]

2- Discriminative genes for each subset reported in Villani et al. [2]

Supplementary Table 7: List of genes resulting from pathway analysis reported in Supplementary Fig. 4c

Upregulated *in vitro* vs *blood*

p-value	q-value	pathway	source	external_id	members_input_overlap	size
5.44E-12	1.13E-09	Electron Transport Chain	Wikipathways	WP111	COX5A; COX5B; NDUFC1; UQCR10; UQCR11; NDUFB3; NDUFB1; UQCRQ; UQCRH; COX6B1; COX7A2; NDUFAB1; NDUFV1; MT-ND6; COX8A; NDUFB6; COX6C; NDUFA7; NDUFA3; NDUFA1; NDUFA8; SLC25A4; UCP2; NDUFS6; COX7B; COX7C; COX17	103
3.72E-07	3.67E-05	mRNA Processing	Wikipathways	WP411	NCBP2; SF3B4; PPM1G; SRSF1; U2AF1; SRSF9; HNRNPU; HNRNPK; HNRNPA2B1; HNRNPAB; SMC1A; PABPN1; PRMT2; SNRPF; SNRPG; SNRPE; SNRPD3; SNRPD2; SNRPD1; METTL3; CSTF2T; RBMX; YBX1	127
5.30E-07	3.67E-05	Pyrimidine metabolism	Wikipathways	WP4022	ZNRD1; NME1; NME2; NME3; DUT; NT5C; POLR3K; POLR2H; TYMS; POLR2G; POLD4; RRM2; TK1; POLR2L; POLR2I; CMPK2; POLR2K; POLR2J	84
5.81E-06	3.02E-04	Retinoblastoma (RB) in Cancer	Wikipathways	WP2446	PCNA; SMC1A; STMN1; TYMS; PRMT2; HMGB2; RBBP7; H2AFZ; MCM7; CDK1; CDK2; CDK4; RRM2; MCM3; MYC; WEE1; TOP2A	89
4.28E-05	1.78E-03	Cell Cycle	Wikipathways	WP179	MAD2L2; PCNA; MAD2L1; MCM5; BUB3; MPEG1; MCM3; YWHAH; MCM7; CDK1; CDK2; CDK4; YWHAB; PTTG1; WEE1; SMC1A; YWHAG	103
8.53E-05	2.63E-03	TYROBP Causal Network	Wikipathways	WP3945	IL18; ITGAX; IL10RA; CD4; RNASE6; TYROBP; IGSF6; ZFP36L2; CD37; GPX1; RUNX3; GAPT	60
8.84E-05	2.63E-03	Regulation of sister chromatid separation at the metaphase-anaphase transition	Wikipathways	WP4240	SMC1A; MAD2L1; BUB3; RAD21; ANAPC11; PTTG1	15
1.98E-04	5.15E-03	Pathogenic Escherichia coli infection	Wikipathways	WP2272	ACTG1; ARPC3; TUBB4B; TUBB; ARPC5; ARPC4; NCL; LY96; RHOA; WAS; ACTB	56
4.33E-04	1.00E-02	Oxidative phosphorylation	Wikipathways	WP623	NDUFAB1; NDUFC1; NDUFV1; NDUFA3; GZMB; NDUFA7; NDUFB6; MT-ND6; NDUFB1; NDUFA8; NDUFS6	61
5.40E-04	1.12E-02	Mitotic G1-G1-S phases	Wikipathways	WP1858	PCNA; TYMS; CDK1; RRM2; TK1; TOP2A	20

Upregulated blood vs *in vitro*

p-value	q-value	pathway	source	external_id	members_input_overlap	size
7.32E-67	3.43E-64	Cytoplasmic Ribosomal Proteins	Wikipathways	WP477	RPS13; RPS12; RPS11; RPS10; RPS17; RPS16; RPL23A; RPS14; RPL6; RPL7; RPL4; RPS18; RPL18A; RPL3; RPS15A; RPS7; RPS6; RPS5; RPS25; RPS3; RPS2; RPL41; RPS8; RPL7A; RPL29; RPS15; RPL24; RPL27; RPL26; RPL21; RPL23; FAU; RPS26; RPL13A; RPS9; RPLP2; RPS3A; RPL8; RPL27A; RPL36A; RPS27; RPS24; RPL5; RPS23; RPS20; RPS21; RPLP1; RPS29; RPL14; RPL15; RPL10; RPL11; RPL9; RPL13; RPS27A; RPL18; RPL19; RPSA; RPLP0; RPL37; RPL34; RPL35; RPL32; RPL30; RPL31; UBA52; RPL10A; RPL12	88
3.55E-12	2.38E-10	Electron Transport Chain	Wikipathways	WP111	MT-CO1; MT-CO2; MT-CO3; NDUFB8; NDUFB7; MT-ND1; MT-ND2; MT-ND3; MT-ND4; MT-ND5; MT-ND4L; NDUFB10; MT-ATP8; SURF1; MT-ATP6; NDUFA6; NDUFA4; MT-CYB; SLC25A6; SLC25A5; COX4I1; COX7A1; COX6A1; NDUFS5; NDUFS7; NDUFS8; UQCRFS1; UQCRC1	103
1.52E-10	4.47E-09	mRNA Processing	Wikipathways	WP411	RBM5; FUS; SF3B5; PRPF4B; NXF1; SRSF10; HNRNPH2; TMED10; SRSF5; SRSF7; SRSF3; SRSF2; SNRPA1; RBM17; PCBP2; HNRNPC; RBM39; TRA2B; LSM7; RNPS1; HNRNPA1; SFPQ; SNRPB; SNRPA; SREK1; DNAJC8; SRRM1; HNRNPD; CLK1	127
1.43E-09	3.95E-08	Translation Factors	Wikipathways	WP107	EIF1; EIF3I; PABPC1; EEF1D; EIF3D; EIF5; EIF6; EEF1B2; ETF1; EIF4A2; EIF4EBP3; EEF1A1; EIF3G; EIF2B1; EIF4A1; EIF2B4; EIF5A	50
2.11E-09	5.51E-08	Proteasome Degradation	Wikipathways	WP183	PSMD8; HLA-A; PSMB10; HLA-C; PSMD4; PSMD7; UBC; UBE2D3; HLA-E; PSME1; UBB; PSMB1; PSMB9; HLA-B; PSMB6; PSMB3; PSMC5; PSMC6; HIST1H2AB	64
4.30E-08	8.77E-07	Allograft Rejection	Wikipathways	WP2328	TUBA1B; HLA-DRA; HLA-DPB1; HLA-C; CXCL8; HLA-A; HLA-DRB1; HLA-E; HLA-DRB5; TNF; HLA-DQB1; C1QA; HLA-DOB; HLA-B; HLA-DPA1; IL1B; VIM; HLA-DMB; HLA-DQA1; HLA-DQA2; HLA-DMA	91
2.68E-07	4.65E-06	Oxidative phosphorylation	Wikipathways	WP623	NDUFA6; MT-ND1; NDUFA4; MT-ND3; MT-ND4; MT-ND5; NDUFB8; NDUFB7; MT-ND2; MT-ND4L; NDUFS5; NDUFS7; NDUFS8; MT-ATP6; NDUFB10; NDUFA11	61
9.61E-06	1.19E-04	miR-targeted genes in leukocytes - TarBase	Wikipathways	WP2003	ATP6V1F; USP1; MCL1; MATR3; TMED10; CEBPB; MAP3K8; RAB34; TUBA1A; TPM3; THBS1; TNFSF9; ARHGDIA; ANXA2; SLC25A1; GOLGA7; HMGA1; RHOG; TRAM1; DDX5; GNA13; BCL6; PPIB; SRSF10	154
1.36E-05	1.60E-04	Photodynamic therapy-induced unfolded protein response	Wikipathways	WP3613	DNAJB9; DDIT3; BCL2L11; HSPA5; CALR; PPP1R15A; DNAJB11; ATF4; XBP1	27
2.76E-05	3.08E-04	Gene and protein expression by JAK-STAT signaling after Interleukin-12 stimulation	Wikipathways	WP4100	ANXA2; SOD2; CNN2; HSPA9; RPLP0; SNRPA1; AIP; TCP1; CFL1; ARF1	36

Note:

Pathway analysis was performed using ConsensusPathDB (cpdb.molgen.mpg.de) [3] *and statistical significance was calculated using the hypergeometric test*

Supplementary Table 8: List of genes resulting from pathway analysis reported in Supplementary Fig. 7c

Upregulated *in vivo* vs *blood*						
**p-value**	**q-value**	**pathway**	**source**	**external_id**	**members_input_overlap**	**size**
7.41E-38	1.43E-35	Retinoblastoma Gene in Cancer	Wikipathways	WP2446	STMN1; DHFR; HMGB2; PLK4; RFC3; RRM2; RRM1; CDC7; FANCG; SKP2; TYMS; CCNE2; SMC2; CDT1; KIF4A; TTK; RFC5; POLD3; PRIM1; RFC4; MYC; TOP2A; PCNA; BARD1; CDC25B; CDC25A; ANLN; ORC1; MCM7; MCM6; MCM4; MCM3; MSH6; RPA3; WEE1; CCNB2; CCNB1; E2F2; E2F1; H2AFZ; CDK1; CDK2; CDK4; POLE2; CDC45; CHEK1; CCNA2	89
6.46E-24	6.23E-22	DNA Replication	Wikipathways	WP466	POLA2; RFC5; RFC4; RFC3; RFC2; GMNN; CDC6; CDC7; CDT1; POLD1; POLD3; PRIM1; PCNA; MCM10; ORC6; ORC5; ORC1; MCM7; MCM6; MCM4; MCM3; MCM2; RPA3; CDK2; POLE2; CDC45	42
6.45E-22	4.15E-20	Cell Cycle	Wikipathways	WP179	PLK1; PKMYT1; CDC6; CDC7; SKP2; CCNA1; CCNE2; TTK; CDC25C; ESPL1; PTTG1; MYC; CHEK1; PCNA; BUB1; RBL1; BUB3; ORC6; CDC25B; CDC25A; ORC5; ORC1; MCM7; MCM6; MCM4; MCM3; MCM2; CCNA2; WEE1; CCNB2; CCNB1; E2F2; E2F1; CDC23; CDC20; CDK1; CDK2; CDK4; CDC45	120
5.76E-20	2.78E-18	Mitotic G1-G1-S phases	Wikipathways	WP1858	PCNA; CDC45; CCNA2; RBL1; E2F1; TYMS; DHFR; CDC25A; CCNA1; CDT1; ORC1; CDK1; FBXO5; TK1; RRM2; CDC6; TOP2A	20
1.09E-16	4.23E-15	G1 to S cell cycle control	Wikipathways	WP45	POLA2; CCNA1; CCNE2; PRIM1; MYC; PCNA; ORC6; CDC25A; ORC5; ORC1; MCM7; MCM6; MCM4; MCM3; MCM2; WEE1; CCNB1; E2F2; E2F1; RPA3; CDK1; CDK2; CDK4; POLE2; CDC45	64
3.33E-10	1.07E-08	Gastric Cancer Network 1	Wikipathways	WP2361	E2F7; TPX2; LIN9; ECT2; CCNA1; CENPF; UBE2C; S100P; KIF15; MCM4; ACTL6A; AURKA; TOP2A	29
1.71E-07	4.71E-06	Regulation of sister chromatid separation at the metaphase-anaphase transitic	Wikipathways	WP4240	MAD2L1; BUB1; BUB3; BUB1B; CENPE; CDC20; ESPL1; PTTG1	15
3.02E-07	7.28E-06	Mitotic G2-G2-M phases	Wikipathways	WP1859	PLK1; CCNB2; CCNB1; CDC25A; CENPF	5
1.10E-06	2.36E-05	DNA Mismatch Repair	Wikipathways	WP531	PCNA; MSH2; EXO1; MSH6; POLD1; LIG1	9
2.64E-06	5.09E-05	Transcriptional Regulation by E2F6	Wikipathways	WP4413	BRCA1; E2F1; RRM2; RAD51; CBX5; CDC7	10

Upregulated *blood* vs *in vivo*						
**p-value**	**q-value**	**pathway**	**source**	**external_id**	**members_input_overlap**	**size**
9.94E-08	1.62E-05	RNA polymerase II transcribes snRNA genes	Wikipathways	WP3827	RNU1-1; RNU4-1; RNU4ATAC; RNU12; RNU5A-1	7
1.57E-06	1.28E-04	Histone Modifications	Wikipathways	WP2369	HIST1H4B; HIST1H4A; HIST1H4F; HIST1H4E; SETD7; HIST2H3D; H3F3B; HIST1H3D; HIST1H3H; HIST1H3A	66
7.41E-05	4.03E-03	Genotoxicity pathway	Wikipathways	WP4286	HIST1H3D; GADD45A; HIST1H2BC; HIST1H2BG; HIST1H2BB; HIST1H1E; HIST1H2BM; HIST1H2BI	64
2.17E-04	8.82E-03	Oxidative Damage	Wikipathways	WP3941	MAP3K9; GADD45A; C1QA; CASP9; C1S; C1R	40
7.52E-04	2.45E-02	Vitamin D Receptor Pathway	Wikipathways	WP2877	G0S2; SEMA3B; IRF4; NINJ1; CLMN; ID1; ABCB1; VDR; MYO9B; GADD45A; MXD1; CYP27B1	184
1.07E-03	2.92E-02	Chromosomal and microsatellite instability in colorectal cancer	Wikipathways	WP4216	BCL2L11; GADD45A; GADD45B; JUN; TCF7L1; CASP9; BBC3	73
3.88E-03	7.71E-02	Exercise-induced Circadian Regulation	Wikipathways	WP410	G0S2; TOB1; NR1D2; PER1; KLF9	48
4.06E-03	7.71E-02	ErbB Signaling Pathway	Wikipathways	WP673	AREG; BCL2L11; SRC; JUN; CAMK2A; GAB1; HBEGF	92
4.64E-03	7.71E-02	Photodynamic therapy-induced AP-1 survival signaling.	Wikipathways	WP3611	MAP2K3; HBEGF; BCL2L11; BMF; JUN	50
4.73E-03	7.71E-02	Amplification and Expansion of Oncogenic Pathways as Metastatic Traits	Wikipathways	WP3678	SRC; JAG1; TCF7L1	16

Note:

Pathway analysis was performed using ConsensusPathDB (cpdb.molgen.mpg.de) [3] *and statistical significance was calculated using the hypergeometric test*

Supplementary Table 9: List of genes resulting from pathway analysis reported in Supplementary Fig. 7d

p-value	q-value	pathway	source	external_id	members_input_overlap	size
**Upregulated *in vitro DC2* vs *in vivo DC2***						
**p-value**	**q-value**	**pathway**	**source**	**external_id**	**members_input_overlap**	**size**
9.55E-15	2.36E-12	Interferon alpha-beta signaling	Wikipathways	WP1835	IFITM3; IFITM2; XAF1; IFI6; GBP2; IFI27; IFITM1; RSAD2; ISG15; BST2; ISG20; IFIT3; IFIT2; IFIT1	21
1.22E-06	1.50E-04	Interleukin-4 and Interleukin-13 signaling	Wikipathways	WP4066	ITGAX; CCL2; TNFRSF1B; STAT1; PIM1; CCND1; S1PR1; HMOX1; FOXO1; VEGFA; VCAM1; ICAM1; MMP9; SOCS1; CCL22; ZEB1; MMP3	97
2.79E-06	2.13E-04	The human immune response to tuberculosis	Wikipathways	WP4197	IFITM1; SOCS1; STAT1; IRF9; MX1; OAS1; IFIT3; IFIT1	22
3.45E-06	2.13E-04	Type II interferon signaling (IFNG)	Wikipathways	WP619	SOCS1; STAT1; IFI6; IRF9; GBP1; EIF2AK2; OAS1; ISG15; ICAM1; IFIT2	37
1.12E-05	5.53E-04	Allograft Rejection	Wikipathways	WP2328	IL2RA; PECR; STAT1; GZMB; CTLA4; CCL19; C1QC; C1QB; C1QA; CD40; COL5A1; VEGFA; HLA-DOB; CD80; CXCL13	91
3.42E-05	1.41E-03	Interleukin-10 signaling	Wikipathways	WP4063	CCL2; TNFRSF1B; CCL19; CCR1; ICAM1; CD80; CCL20; CCL22; IL1R1	38
6.13E-05	2.16E-03	IL1 and megakaryocytes in obesity	Wikipathways	WP2865	CCL2; F2R; PLA2G7; CCR3; ICAM1; MMP9; IL1R1	24
1.23E-04	3.79E-03	Photodynamic therapy-induced NF-kB survival signaling	Wikipathways	WP3617	BCL2A1; CCND1; BIRC3; VEGFA; VCAM1; ICAM1; MMP9; MMP3	35
2.56E-04	6.26E-03	Complement and Coagulation Cascades	Wikipathways	WP558	CFH; CFI; C1QC; C1QB; C1QA; PLAT; F2R; SERPING1; TFPI; A2M	59
2.96E-04	6.26E-03	Dengue-2 Interactions with Complement and Coagulation Cascades	Wikipathways	WP3896	CFH; CFI; C1QC; C1QB; C1QA; PLAT; F2R; SERPING1; TFPI; A2M	60
**Upregulated *in vivo DC2* vs *in vitro DC2***						
**p-value**	**q-value**	**pathway**	**source**	**external_id**	**members_input_overlap**	**size**
1.76E-05	3.16E-03	Spinal Cord Injury	Wikipathways	WP2431	CXCL1; PTGS2; CXCL2; CSPG4; FOS; CXCL8; NR4A1; VCAN; ZFP36; ANXA1; ARG1; RHOB; AQP1	117
6.44E-05	5.79E-03	Interleukin-4 and Interleukin-13 signaling	Wikipathways	WP4066	ANXA1; PTGS2; LIF; FOS; CXCL8; BIRC5; RHOU; OSM; CD36; COL1A2; BCL2	97
5.18E-04	3.11E-02	Interleukin-10 signaling	Wikipathways	WP4063	CXCL1; PTGS2; LIF; CXCL2; IL1RN; CXCL8	38
6.97E-04	3.14E-02	Retinoblastoma Gene in Cancer	Wikipathways	WP2446	CCNB2; STMN1; E2F2; DHFR; CDC25B; MCM7; HMGB2; CDC7; CHEK1	89
1.60E-03	4.66E-02	White fat cell differentiation	Wikipathways	WP4149	EGR2; WNT10B; KLF4; GATA2; KLF2	32
1.62E-03	4.66E-02	Cell Cycle	Wikipathways	WP179	CCNB2; TGFB2; E2F2; CCNA1; CDC25B; CDC20; MCM7; CDC7; CDC6; CHEK1	120
1.86E-03	4.66E-02	Mitotic G1-G1-S phases	Wikipathways	WP1858	DHFR; MYBL2; CDC6; CCNA1	20
2.07E-03	4.66E-02	VEGFA-VEGFR2 Signaling Pathway	Wikipathways	WP3888	ANXA1; PTGS2; ITGB5; NR4A2; NR4A3; NR4A1; CAPN2; HBEGF; ETS1; PXN; CXCL8; EGR3; BCL2; PBK; IGFBP7	236
4.28E-03	7.66E-02	Intrinsic Pathway for Apoptosis	Wikipathways	WP1841	PMAIP1; BCL2	4
4.58E-03	7.66E-02	Vitamin D Receptor Pathway	Wikipathways	WP2877	TGFB2; CAMP; S100A9; BTLA; IRF8; ADRB2; KLF4; TREM1; S100A8; S100A4; S100A6; STEAP4	184

Note:

Pathway analysis was performed using ConsensusPathDB (cpdb.molgen.mpg.de) [3] and statistical significance was calculated using the hypergeometric test

References

1. McGovern, N. et al. Human dermal CD14(+) cells are a transient population of monocyte-derived macrophages. *Immunity* **41**, 465-477 (2014).
2. Villani, A.C. et al. Single-cell RNA-seq reveals new types of human blood dendritic cells, monocytes, and progenitors. *Science* **356** (2017).
3. Kamburov, A., Wierling, C., Lehrach, H. & Herwig, R. ConsensusPathDB--a database for integrating human functional interaction networks. *Nucleic Acids Res* **37**, D623-628 (2009).
