# Supplementary material for: Engineered niches support the development of human dendritic cells in humanized mice
Source: Nat Commun. 2020 Apr 28;11:2054. doi: 10.1038/s41467-020-15937-y (PMC7189247; doi:10.1038/s41467-020-15937-y)
Supplement: Supplementary file 2 — Reporting Summary [file 41467_2020_15937_MOESM2_ESM.pdf]

nature research

Corresponding author(s): Pierre Guermonprez

Last updated by author(s): Mar 12, 2020

Reporting Summary

Nature Research wishes to improve the reproducibility of the work that we publish. This form provides structure for consistency and transparency in reporting. For further information on Nature Research policies, see Authors & Referees and the Editorial Policy Checklist.

Statistics

For all statistical analyses, confirm that the following items are present in the figure legend, table legend, main text, or Methods section.

n/a	Confirmed	
☐	☒	The exact sample size (n) for each experimental group/condition, given as a discrete number and unit of measurement
☐	☒	A statement on whether measurements were taken from distinct samples or whether the same sample was measured repeatedly
☐	☒	The statistical test(s) used AND whether they are one- or two-sided<br>*Only common tests should be described solely by name; describe more complex techniques in the Methods section.*
☒	☐	A description of all covariates tested
☐	☒	A description of any assumptions or corrections, such as tests of normality and adjustment for multiple comparisons
☐	☒	A full description of the statistical parameters including central tendency (e.g. means) or other basic estimates (e.g. regression coefficient) AND variation (e.g. standard deviation) or associated estimates of uncertainty (e.g. confidence intervals)
☒	☐	For null hypothesis testing, the test statistic (e.g. F, t, r) with confidence intervals, effect sizes, degrees of freedom and P value noted<br>*Give P values as exact values whenever suitable.*
☒	☐	For Bayesian analysis, information on the choice of priors and Markov chain Monte Carlo settings
☒	☐	For hierarchical and complex designs, identification of the appropriate level for tests and full reporting of outcomes
☒	☐	Estimates of effect sizes (e.g. Cohen's d, Pearson's r), indicating how they were calculated

Our web collection on statistics for biologists contains articles on many of the points above.

Software and code

Policy information about availability of computer code

Data collection	BD FACSDiva<br>Cytof Software (Illumina)<br>Leica Application Suite
Data analysis	FlowJo (TreeStar, version 10.2)<br>Graphpad Prism (version 8.0)<br>Rstudio (1.2.5001)<br>ImageJ (1.49c)<br>BubbleGum (v1.3.19)<br>Partek Flow (Partek Inc., build 7.0.18.0514)<br>Morpheus (https://software.broadinstitute.org/morpheus)<br>FastQC (version 0.11.19)<br>Hisat2 (version 2.1.0)<br>STAR Aligner (v2.5.3a)<br>R (version 3.4.4)<br>R package DESeq2 (version 1.24.0)<br>R package ggplot2 (version 3.2.1)<br>UMAP (2.4.0)<br>Python (3.6)<br>Galaxy (version 18.05.rc1)

For manuscripts utilizing custom algorithms or software that are central to the research but not yet described in published literature, software must be made available to editors/reviewers. We strongly encourage code deposition in a community repository (e.g. GitHub). See the Nature Research guidelines for submitting code & software for further information.

Data

Policy information about availability of data

All manuscripts must include a data availability statement. This statement should provide the following information, where applicable:

- Accession codes, unique identifiers, or web links for publicly available datasets
- A list of figures that have associated raw data
- A description of any restrictions on data availability

Data that support the findings of this study have been deposited in Gene Expression Omnibus (GEO) with the accession codes GEO144435 and GEO145803

Field-specific reporting

Please select the one below that is the best fit for your research. If you are not sure, read the appropriate sections before making your selection.

☒ Life sciences ☐ Behavioural & social sciences ☐ Ecological, evolutionary & environmental sciences

For a reference copy of the document with all sections, see nature.com/documents/nr-reporting-summary-flat.pdf

Life sciences study design

All studies must disclose on these points even when the disclosure is negative.

Sample size	For both in vitro and in vivo experiments, at least 3 independent donors were used to account for biological variation of cord blood samples (as previously reported by Doulatov et al., Nat Immunol. 2010 Jul;11(7):585-93 and van Galen et al., Cell Stem Cell. 2014 Jan 2;14(1):94-106). For in vivo experiments, at least 3 mice/group were used.
Data exclusions	No data were excluded from analysis.
Replication	All the experiments supporting major conclusions were preformed at least 2 times to account for technical variations. All attempts of replication were successful.
Randomization	In each in vivo experiment, animals of the same age and background (NSG) were randomly assigned to each group. In experiments performed in vitro, each cord blood donor was split and allocated to both control and tested conditions (eMSCs).
Blinding	Investigators were not blinded to group allocation. Blinding was not relevant for this study since differences between groups were determined by the physical measurement of cell frequency within each sample by flow cytometry

Reporting for specific materials, systems and methods

We require information from authors about some types of materials, experimental systems and methods used in many studies. Here, indicate whether each material, system or method listed is relevant to your study. If you are not sure if a list item applies to your research, read the appropriate section before selecting a response.

Materials & experimental systems

n/a	Involved in the study	
☐	☒	Antibodies
☐	☒	Eukaryotic cell lines
☒	☐	Palaeontology
☐	☒	Animals and other organisms
☐	☒	Human research participants
☒	☐	Clinical data

Methods

n/a	Involved in the study	
☒	☐	ChIP-seq
☐	☒	Flow cytometry
☒	☐	MRI-based neuroimaging

Antibodies

Antibodies used	Antibodies used in Flow Cytometry (target, fluorochrome, clone, supplier, cat.#):<br>huCD45 APC-Cy7/APC HI30 Biolegend 304014 304012<br>muCD45 PerCP-Cy5.5 104 eBiosciences 45-0454-82<br>muCD31 A647 MEC13.3 Biolegend 102516<br>CD14 Pe-Cy7/BV786/APC HCD14/M5E2 Biolegend 325618 301840 325608<br>CD16 BV421 3G8 Biolegend 302038<br>CD141 PE/PE-Cy7/PerCP-Cy5.5 M80 Biolegend 344104 344110 344112<br>CD1c FITC/PE-Cy7/APC L161 Biolegend 331518 331516 331524<br>Clec9A PE/APC 8F9 Biolegend 353804 353806<br>HLA-DR BV510 L243 Biolegend 307646

CD123 PerCP-Cy5.5/APC/PE_Dazzle 6H6 Biolegend 306016 306012 306034
CD303 APC 201A Biolegend 354206
CD304 APC 12C2 Biolegend 354506
CD163 FITC/BV711 GHI/61 Biolegend 333618 333630
CD206 PE/APC/PerCP-Cy5.5 15.2/6H6 Biolegend 321106 321110 321122
CD3 BV786/FITC OKT3 Biolegend 317330 317306
CD327/Siglec6 FITC 767329 R&D FAB2859G
Axl PE/FITC 108724 R&D FAB164P
CD15 BV510/BV421 W6D3 Biolegend 323028 323040
CD19 PE HIB19 Biolegend 302208
CD34 APC/FITC 561 Biolegend 343608 343604
CD45RA BV510/PE HI100 Biolegend 304142 304108
a-tmSCF biotin polyclonal R&D AF-255-NA
a-Flt3L biotin polyclonal R&D AF-308-NA
CD1a APC/FITC/Percp-Cy5.5 HI149 Biolegend 300110 300104 300130
NKp46 biotin 9E2 Biolegend 331906
CD3 biotin OKT3 Biolegend 317320
CD19 biotin HIB19 Biolegend 302204
CD20 biotin 2H7 Biolegend 302350
CD15 biotin MC-480 Biolegend 125604
CD203c biotin FR3-16A11 Miltenyi Biotec 130-092-345
Streptavidin APC-Cy7 - Biolegend 405208
IFNa PE 7N4-1 BD Bioscience 560097
IL-12 PE C11.5 BD Bioscience 559329
TNFa AF700 SE5A5 Biolegend 502928
CD4 APC-Cy7 A161A1 Biolegend 357416
CD8 APC HIT8a Biolegend 300912
CD45RO PerCP-Cy5.5 UCHL1 Biolegend 304252
CD86 PE BU63 Biolegend 374206
CD83 FITC HB15e Biolegend 305306

Antibodies used in Immunofluorescence (target, fluorochrome, clone, supplier, cat.#):
murine CD31 A647 MEC13.3 Biolegend 102516
CD1c PE L161 Biolegend 331506
Clec9A PE 8F9 Biolegend 353804
CD34 APC 561 Biolegend 343608
CD45 purified HI30 Biolegend 304002
CD45 APC HI30 Biolegend 304037
anti-mouse Cy3 polyclonal Jackson Immunoresearch 115-165-166
anti-mouse Cy5 polyclonal Jackson Immunoresearch 115-175-166

Neutralizing antibodies (target, fluorochrome, clone, supplier, cat.#)::
human GM-CSF unconjugated polyclonal R&D AF-215-SP
Goat IgG unconjugated polyclonal R&D AB-108-C

Antibodies used in Cytof (target, metal, clone, supplier, cat.#):
CD45 Y89Di HI30 Fluidigm 3089003B
CD14 Cd112Di TuK4 Invitrogen MHCD1400
CD15 In115Di hi98 Biolegend 301902
Clec12A Pr141Di 50C1 R&D Sysytems MAB2946
CD5 Nd142Di UCHT2 Biolegend C 300602
CD2 Nd143Di RPA-2.10 Biolegend 300202
CD64 Nd144Di 10.1 Biolegend 305002
CD68 Nd145Di Y1/82A eBioscience 14-0688-80
CD19 Nd146Di HIB19 eBioscience 14-0199-82
CD20 Nd146Di 2h7 eBioscience 14-0209-82
CD3 Nd146Di ucht1 Biolegend 317302
CD26 Sm147Di BA5B Biolegend 302702
CD45RA Nd148Di HI100 Biolegend 304102
HLA-DR Sm149Di L243 Biolegend 307602
CD80 Nd150Di L307.4 BD Biosciences 557223
CADM1 Eu151Di 3E1 MBL CM004-3
CD1c Sm152Di L161 Biolegend 331502
FcER1 Eu153Di AER-37 eBioscience 14-5899-82
CD327 Sm154Di 767329 R&D systems MAB2859
CD33 Gd155Di wm53 BD Biosciences 555449
CD163 Gd156Di GHI/61 Biolegend 333602
CCR2 Gd157Di k036c2 Biolegend 357202
CD56 Gd158Di NCAM16.2 BD Biosciences 559043
SLAN Tb159Di DD-1 Miltenyi Biotec 130-093-031
CD207 Gd160Di DCGM4/122D5 Novus Biologicals DDX0363P-100
CD172a Dy161Di SE5A5 Biolegend 323902
AXL Dy162Di MM0098-2N33 Novus Biologicals MM0098-2N33
CD123 Dy163Di 6h6 BD Biosciences 554527
CD303 Ho165Di 201A Biolegend 354202

	CD86 Er166Di IT2.2 BD Biosciences 555663<br>OX40L Er167Di 11C3.1 Biolegend 326302<br>CCR7 Er168Di mab197 R&D systems MAB197-100<br>LYVE1 Tm169Di af2089 R&D systems AF2089<br>CD88 Er170Di S5/1 Biolegend 344302<br>CD34 Yb171Di 581 Biolegend 343502<br>TSLPR Yb172Di 1B4 Biolegend 322802<br>CX3CR1 Yb173Di K0124E1 Biolegend 355702<br>CD206 Yb174Di 15.2 Biolegend 321102<br>PDL1 Lu175Di 29E.2A3 Biolegend 329719<br>CD11b Yb176Di ICRF44 Biolegend 301302<br>DNA Ir191Di<br>DNA Ir193Di<br>Cisplatin Pt195Di<br>CD16 Bi209Di 3G8 Biolegend 302002
Validation	All antibodies were used according to manufacturer's instructions available at:<br>Biolegend (www.biolegend.com)<br>eBioscience (www.thermofisher.com)<br>R&D Systems (www.rndsystems.com)<br>Jackson ImmunoResearch (www.jacksonimmuno.com)<br>Working dilutions were identified by testing the antibodies on human freshly isolated PBMCs.

Eukaryotic cell lines

Policy information about cell lines

Cell line source(s)	The murine bone marrow-derived stromal cell line MS5 was kindly provided by Dr Julie Helft (Institut Curie, Paris, France).<br>MS5 cells were originally purchased from DSMZ (no. ACC 441).<br>OP9 bone marrow-derived stromal cells were purchased from ATCC (no. CRL-2749)
Authentication	The cell lines used were not authenticated.
Mycoplasma contamination	The cell lines used in this study (MS5, OP9 and engineered stromal cells) were regularly tested and resulted negative for mycoplasma contamination.
Commonly misidentified lines<br>(See ICLAC register)	no commonly misidentified lined were used in the study.

Animals and other organisms

Policy information about studies involving animals; ARRIVE guidelines recommended for reporting animal research

Laboratory animals	All in vivo experiments were performed using NOD.Cg-Prdcscid Il2rgtm1Wjl/SzJ (NSG) mice (JAX #005557). Both males and females were used between 8 and 12 weeks of age. Mice were maintained in specific-pathogen-free conditions and handled according to protocols approved by the UK Home Office.
Wild animals	This study did not involve wild animals.
Field-collected samples	This study did not involve samples collected from the field.
Ethics oversight	UK Home Office PPL 70/8278 "ANTIGEN PRESENTATION BY DENDRITIC CELLS" 2014

Note that full information on the approval of the study protocol must also be provided in the manuscript.

Human research participants

Policy information about studies involving human research participants

Population characteristics	Umbilical Cord Blood samples were donated to Anthony Nolan Cord Blood Bank and Cell Therapy Centre. No relevant population characteristics are available.
Recruitment	Umbilical Cord Blood samples were collected and supplied by Anthony Nolan Cord Blood Bank and Cell Therapy Centre upon informed consent acquisition
Ethics oversight	Research was conducted under Material Transfer Agreement (MTA) with Anthony Nolan Cord Blood Bank and Cell Therapy Centre

Note that full information on the approval of the study protocol must also be provided in the manuscript.

Flow Cytometry

Plots

Confirm that:

☒ The axis labels state the marker and fluorochrome used (e.g. CD4-FITC).

☒ The axis scales are clearly visible. Include numbers along axes only for bottom left plot of group (a 'group' is an analysis of identical markers).

☒ All plots are contour plots with outliers or pseudocolor plots.

☒ A numerical value for number of cells or percentage (with statistics) is provided.

Methodology

Sample preparation	Extracellular staining of cells was preformed by incubating the samples for 30 minutes at 4C in antibody mixes prepared using FACS buffer (PBS, 1% BSA, 2 mM EDTA). For intracellular staining, samples were fixed and permeabilized using the Cytofix/CytopermTM kit (BD Biosciences) according to manufacturers' instructions.
Instrument	LSR Fortessa II (BD Biosciences, BD Diva Software)<br>AriaII (BD Biosciences, BD Diva Software)
Software	FlowJo software (TreeStar, version 10.2)
Cell population abundance	Purity of FACS-sorted samples was assessed at the sorter and frequency of sorted cells was above 95% of live cells.
Gating strategy	The gating strategy used to analyze and FACS-sort human DC subsets are exemplified in Supplementary Fig.2 and Supplementary Fig.8.

☒ Tick this box to confirm that a figure exemplifying the gating strategy is provided in the Supplementary Information.
